# Supplementary material for: Applying a genetic risk score model to enhance prediction of future multiple sclerosis diagnosis at first presentation with optic neuritis
Source: Nat Commun. 2024 Feb 28;15:1415. doi: 10.1038/s41467-024-44917-9 (PMC10902342; doi:10.1038/s41467-024-44917-9)
Supplement: Supplementary file 1 — Supplementary Information [file 41467_2024_44917_MOESM1_ESM.pdf]

Applying a genetic risk score model to enhance prediction of future multiple sclerosis diagnosis at first presentation with optic neuritis

1. Supplementary Methods

1.1. Source of diagnosis based on diagnostic codes in UKBB

1.2. Generating MS-GRS

1.3. External validation: Geisinger, USA

1.3.1. Data Source and Population

1.3.2. Source of diagnosis based on diagnostic codes

1.3.3. Generating the MS-GRS

1.4. External validation: FinnGen, Finland

1.4.1. Data Source and Population

1.4.2. Source of diagnosis based on diagnostic codes

1.4.3. Generating the MS-GRS

2. Supplementary Results

2.1. External Validation Results in Geisinger (USA, PA) and FinnGen (Finland)

2.1.1. MS-GRS was discriminative of MS

2.1.2. Genetic overlap of MS, ON and MS associated ON

2.1.3. Validating UKBB model of MS-free survival in undifferentiated ON

2.2. Supplementary UKBB Demographic Data

2.2.1. Additional UKBB-specific variables (Vitamin D, Country of Birth, Townsend Deprivation Index) & Tables for all ON and MS cases

2.2.2. Age at diagnosis of MS

2.2.3. Age at diagnosis of ON

2.2.4. Age at diagnosis of ON in undifferentiated ON

2.2.5. Causes of death in MS & ON

2.3. MS-GRS in MS-ON: MS first VS ON first across three datasets

2.4. MS-GRS association with other variables:

2.4.1. Age at diagnosis of MS/ON

2.4.2. Sex

2.4.3. Ethnicity

2.5. Unadjusted Kaplan-Meier of MS-free survival
2.6. Cox Proportional Hazard Models
2.6.1. Final model proportional hazard assumptions
2.6.2. Final model hazard ratio within quartiles
2.6.3. Continuous age at diagnosis of ON modelling
2.6.4. Interaction between sex and age at ON diagnosis
2.6.5. Adjusting for age at UKBB enrolment

3. Supplementary Results – Sensitivity Analyses in UK Biobank
3.1. White European population definition
3.1.1. Cases and demographic characteristics
3.1.2. MS-GRS ROC-AUC
3.1.3. Genetic overlap of MS, ON, and MS associated ON
3.1.4. MS-GRS predictive of future MS in individuals with ON
3.2. Summary of other subgroup analyses
3.2.1. Excluding cases diagnosed before 20 years of age
3.2.2. Strict definitions of diagnoses
3.2.3. Summary and comparison of subgroup analyses

4. Full List of Consortia Members
4.1. UK Biobank Eye Health & Vision Consortium
4.2. Geisinger-Regeneron DiscovEHR
4.3. FinnGen

1. Supplementary Methods

1.1 Source of diagnosis based on diagnostic codes

1.1.1 Case Identification

Our case identification process is detailed in **Supplementary Fig. 1**. In brief, we first included the UKBB-defined 'first occurrence' cases of MS and ON. These are centrally generated by UKBB from HES data (using International Classification of Disease version 10 (ICD-10) and ICD-9 codes), primary care records (using Read2 and Read3 codes) and self-report (See Supplementary Table 1).[1,2] Second, in order to verify first-occurrence data, we searched data-fields with self-reported illnesses (self-report codes) and UKBB HES data (ICD-10 & ICD-9) and retrieved an additional 113 MS and 54 ON cases. These cases were all identified from HES data, with no additional cases from 'self-report'. Third, we manually searched primary care data in all participants with missing 'first occurrence data', using additional Read codes identified by our neuro-ophthalmologists (See **Supplementary Table 1**). We identified an additional 5 MS cases and 196 ON cases. Through this process, we identified that where the UKBB 'first occurrence' cases were defined by Read3 codes (rather than Read2 codes), two key ON codes were omitted from capture, namely, F4H3 'Optic Neuritis' (n= 128 added) and F4H32 'Acute retrobulbar neuritis' (n=66 added). Two cases were identified from Read3 code, 'Retrobulbar optic neuritis classified elsewhere' (FyuJ1). Four MS cases were identified from Read2 codes, 'Management of multiple sclerosis in palliative phase' (8Cc4, n=2) and 'Multiple sclerosis multidisciplinary review' (666B, n=2), with the fifth case (F20z) subsequently removed due to missing event date.

1.1.2 **Case Exclusion**

Following code review by our neuro-ophthalmologists, we excluded multiple UKBB codes. These included: 'drug-induced optic neuropathy' (F4H34, n=13); 'Inherited optic neuropathy' (XaE6B, n=0); 'idiopathic optic disc swelling' (XaE6C, n=0); 'infiltrative optic neuropathy' (XaE6E, n=0); 'Optic papillitis' (XaF1F and F4H31, n=7); UKBB code X75hh (n=0), and additional codes not used centrally by UKBB, but which could still appear in data from 'self-report', HES or primary care record data, namely, 'Meningococcal Optic Neuritis' (A36y0, n=2) and 'Syphilitic optic atrophy' (A36y3 and A36y4, n=2).

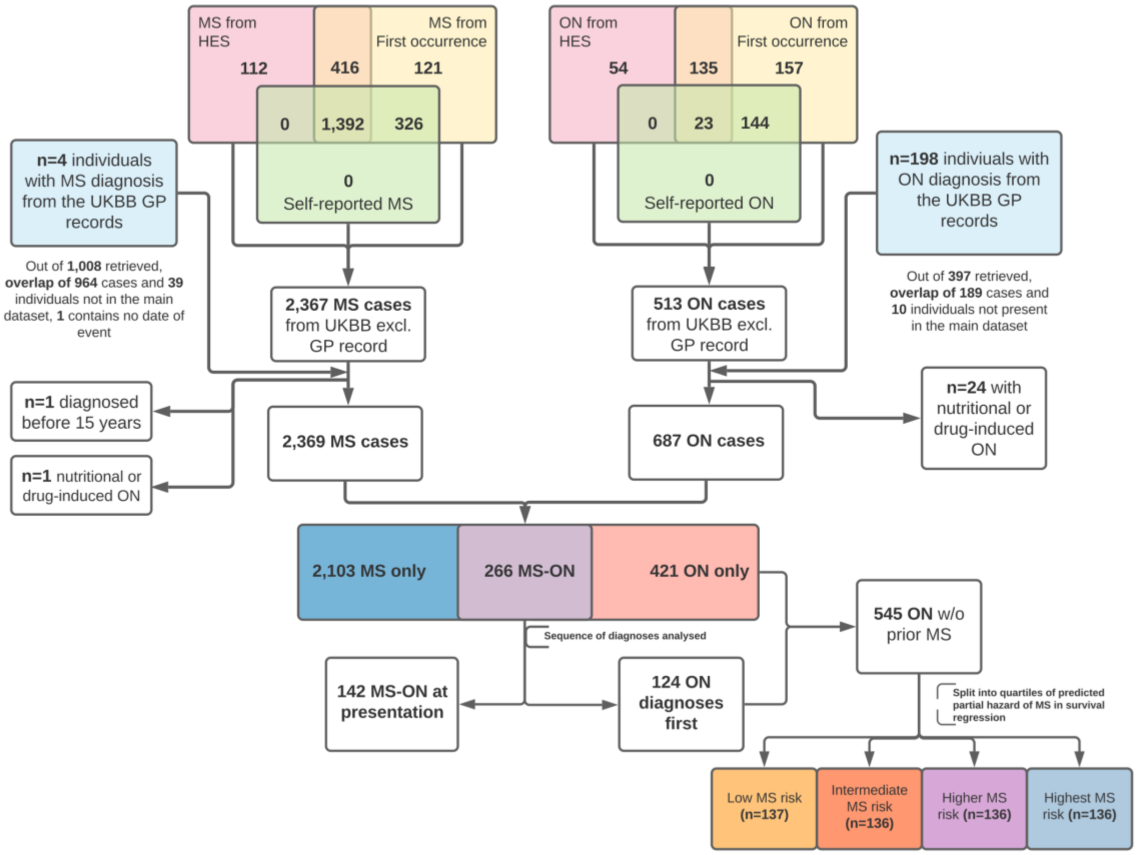

Supplementary Fig. 1. **Extended flow diagram showing the sources of diagnoses for UK Biobank.** Numbers shown after performing exclusions based on missingness, relatedness, quality-control. Abbreviations: MS – Multiple sclerosis, ON – Optic neuritis, HES – Hospital episode statistics, GP records – Primary care data (general practitioner records).

Supplementary Table 1. Case definition of optic neuritis and multiple sclerosis. Based on diagnostic codes used for inclusion and exclusion of participants for UKBB, Geisinger, FinnGen.

		Optic Neuritis	Multiple Sclerosis
UKBB	ICD-10	H46	G35
	ICD-9	377.3	340
	Self-report	1435	1261
	UKBB Read 2 codes	F4H3 Optic neuritis	F20.. Multiple sclerosis
		F4H30 Unspecified optic neuritis	F200. Multiple sclerosis of the brain stem
		F4H3z ON Not otherwise specified	F201. Multiple sclerosis of the spinal cord
		F4H32 Acute retrobulbar neuritis	F203. exacerbation of Multiple sclerosis
	UKBB Read 3 codes	F4H30 Unspecified optic neuritis	F20.. Multiple sclerosis
		F4H3z ON Not otherwise specified	F200. Multiple sclerosis of the brain stem
			F201. Multiple sclerosis of the spinal cord
			F203. exacerbation of Multiple sclerosis
	Additional Read codes included	FyuJ100 Retrobulbar neuritis in diseases classified elsewhere	666B.00 MS MDT Review
		F4H3 Optic Neuritis (in Read3)	8Cc4.00 Management of MS in palliative phase
			-
		F4H32 Acute retrobulbar neuritis (in Read 3)	-
	Codes used for exclusion	F4H31 Optic papillitis	-
		F4H33 Nutritional optic neuropathy	-
		F4H34 Drug induced optic neuropathy	-
		XaE6B Inherited optic neuropathy	-

		XaE6C Idiopathic optic disc swelling	-
		XaE6E Infiltrative optic neuropathy	-
		XaF1F Infiltrative optic neuropathy	-
	Additional codes for exclusion	A36y000 Meningococcal optic neuritis	-
		A94y400 Syphilitic optic atrophy	-
		A94y300 Syphilitic optic atrophy	-
		F4H1200 Post-inflammatory optic atrophy	-
**Geisinger**		**Optic Neuritis**	**Multiple Sclerosis**
	ICD-10	H46	G35
	ICD-9	377.3	340
	Codes used for exclusion	H46.00, H46.01, H6.02, H46.03, 377.31 Optic papillitis	-
		H46.2, 377.33 Nutritional optic neuropathy	-
		H46.3, 377.34 Toxic optic neuropathy	
**FinnGen**	**FinnGen endpoint**	**H7_OPTNEURITIS**	**G6_MS**
	Disease full name	Optic neuritis	Multiple Sclerosis
	ICD 10	H46	G35
	ICD 9	377.3	340
	ICD 8	367.0[2-9]	340.99
	Additional FinnGen endpoint included	H7_RETROBULBNEURINOTH	-
	Disease full name	Retrobulbar neuritis in diseases classified elsewhere	-
	ICD 10	H48.1	-
	ICD 9	N/A	-
	ICD 8	N/A	-

Supplementary Table 2- Detailed optic neuritis Read codes description used in our analysis and by UKBB when translating Primary care data to UKBB 'first occurrence'. Diagnostic codes coinciding between data sources are colour coded in green, discrepancies are highlighted in red. In yellow shown are additional codes that were included, and in blue are codes that resulted in participant exclusion. Where Read codes are reported in the first column, they were used both as Read2 and Read3.

**Searched in Primary care linked data**	**Data-coding 1834**	**Data-coding 1835**	**Description**
**Read 2 / Read 3**	Read2 used by UKBB	Read3 (CTV3) used by UKBB	
A36y000			Meningococcal optic neuritis
**F4H3.00**	F4H3.	Absent in UKBB	Optic neuritis (ON)
**F4H3000**	F4H30	F4H30	Unspecified ON
**F4H3z00**	F4H3z	F4H3z	ON not otherwise specified
**F4H3200**	F4H32	Absent in UKBB	Acute retrobulbar neuritis
F4H31	F4H31	F4H31	Optic papillitis
F4H33	F4H33	F4H33	Nutritional optic neuropathy
F4H34	F4H34	F4H34	Drug induced optic neuropathy
A94y400			Syphilitic optic atrophy
**FyuJ100**			Retrobulbar neuritis in diseases classified elsewhere
A94y300			Syphilitic optic atrophy
F4H1200			Post inflammatory optic atrophy
X75hh		X75hh	Corneal nerves inflamed
XaE6B		XaE6B	Inherited optic neuropathy

XaE6C		XaE6C	Idiopathic optic disc swelling
XaE6E		XaE6E	Infiltrative optic neuropathy
XaF1F		XaF1F	Optic papillitis (same as F4H31)
Links to UKBB GP – ICD10 mapping	https://biobank.ctsu.ox.ac.uk/crystal/coding.cgi?id=1834&nl=1	https://biobank.ctsu.ox.ac.uk/crystal/coding.cgi?id=1835&nl=1	

Supplementary Table 3: Multiple sclerosis Read codes description used by us and by UKBB. In the first column, we show all Read codes that were used for searching the UKBB GP records. In the second and third columns, we show codes that were used centrally by the UKBB when translating Primary care linked data to 'first occurrence'

Searched in Primary care linked data	Data-coding 1834	Data-coding 1835	Description
**Read 2 / Read 3 (CTV3)**	Read2 used by UKBB	Read3 (CTV3) used by UKBB	
666A.00			Multiple sclerosis review
666B.00			Multiple sclerosis multidisciplinary review
8Cc0.00			Management of multiple sclerosis in onset phase
8Cc1.00			Management of multiple sclerosis in early disease phase
8Cc2.00			Management of multiple sclerosis in stable disability phase
8Cc4.00			Management of multiple sclerosis in palliative phase
8CS1.00			Multiple sclerosis care plan agreed
8Hkv.00			Referral to community multiple sclerosis team
8IAb.00			Multiple sclerosis review declined
9kG..00			Spec. serv. for pat with multiple sclerosis
9mD..00			Multiple sclerosis monitoring administration
9mD0.00			Multiple sclerosis monitoring first letter
9mD1.00			Multiple sclerosis monitoring second letter
9mD2.00			Multiple sclerosis monitoring telephone invitation
9mD3.00			Multiple sclerosis monitoring third letter
F20..00	F20..	F20..	Multiple sclerosis
F200.00	F200.	F200.	Multiple sclerosis of the brain stem
F201.00	F201.	F201.	Multiple sclerosis of the spinal cord
F202.00	F202.	F202.	Generalised multiple sclerosis
F203.00	F203.	F203.	Exacerbation of multiple sclerosis
F204.00	F204.		Benign multiple sclerosis
F205.	F205.		Malignant multiple sclerosis
F206.00	F206.		Primary progressive multiple sclerosis
F207.00	F207.		Relapsing and remitting multiple sclerosis
F208.00	F208.		Secondary progressive multiple sclerosis
F20z.00	F20z.	F20z.	Multiple sclerosis NOS
ZRVE.00			Kurtzke multiple sclerosis rating scale
F20..11			Disseminated sclerosis
XaP1B		XaP1B	Relapsing and remitting multiple sclerosis
XaOzZ		XaOzZ	Benign multiple sclerosis
XaP0X		XaP0X	Malignant multiple sclerosis
XaP0r		XaP0r	Primary progressive multiple sclerosis
X005d		X005d	Chronic progressive multiple sclerosis
X005c		X005c	Acute relapsing multiple sclerosis
XaP1V		XaP1V	Secondary progressive multiple sclerosis
X005e		X005e	Remittent-progressive multiple sclerosis

1.2 Generating MS-GRS

non-HLA-GRS

First, we extracted 317 autosomal variants associated with MS from summary statistics of IMSGC GWAS meta-analysis discovery phase.[3] This included 200 genome-wide significant variants with P-value < $5*10^{-8}$ (IMSGC 2019 Supplementary Table 7),[3] and 117 strongly suggestive variants with *P*-value > $5*10^{-8}$ and < 10^{-5} (IMSGC 2019 Supplementary Table 14).[3] We excluded all variants from the extended HLA region on chromosome 6 (chr6:25 Mbp to chr6:35 Mbp, hg19), and sex-chromosome variants. Next, in pairs of variants in linkage disequilibrium (r^2 >0.2) we manually removed one variant (n=7). Next, we ensured that palindromic (strand-ambiguous) variants, such A/T or C/G and vice versa were removed (n=1, rs34536443), and removed duplicated variants (n=1, rs6072343). Finally, we removed variants missing from UKBB imputed data (n=1, rs34536443). We did not exclude variants based on either Hardy-Weinberg equilibrium, minor allele frequency (MAF) or imputation quality, as these quality control steps were performed centrally by the UKBB on the imputed genotypes we used, and thus would not have significantly affected the results.[4] **Supplementary Data 1** contains 307 final SNPs included in UKBB MS-GRS, as well as UKBB imputation INFO score, and minor allele frequency for alleles across Geisinger (USA, PA) and FinnGen (Finland) validation datasets.

HLA-GRS

For the HLA-GRS, we used a ten-allele interaction model reported by Moutsianas *et al*.[5] It consisted of eight HLA alleles and two SNPs from the HLA region (29.9 to 33.6 Mbps on chr6 hg19). We captured interactions between the alleles by calculating the interactive model, scoring imputed HLA alleles while employing both additive effects, homozygote correction terms (I_{xHOM}) and conditional scoring of some HLA alleles (I_x), where x is an HLA allele. This is summarised in the equation by Moutsianas *et al*:[5]

$$HLA_{\boldsymbol{GRS}} = G_{DRB1501} * \beta 1 + I_{DRB1501HOM} * \beta 1_{HOM} + G_{A0201} * \beta 2 + I_{A0201HOM} * \beta 2_{HOM} + I_{DRB0301HOM} * \beta 3_{HOM} + G_{DRB1303} * \beta 4 + G_{DRB0801} * \beta 5 + G_{rs9277565} * \beta 6 + G_{B4402} * \beta 7 + G_{B3801} * \beta 8 + G_{DQA0101} * I_{DRB1501} * \beta 9_{INT} + G_{rs2229092} * \beta 10$$

$$where\ G_x \in \{0,1,2\}, I_{xHOM} = \begin{cases} 1, & if\ G_x == 2 \\ 0, & otherwise \end{cases}, I_x = \begin{cases} 1, & if\ G_x \geq 2 \\ 0, & otherwise \end{cases} for\ HLA\ allele\ or\ SNP\ x,$$
$$G_x\ is\ the\ allele\ genotype, and\ \beta\ is\ the\ corresponding\ beta\ coefficient\ (\ln x)$$

The HLA alleles and SNPs and their respective terms used in the model are described (**Supplementary Table 4**). We used HLA alleles imputed centrally by the UKBB,[3] using hard-called alleles, rather than probabilities.

Supplementary Table 4. HLA alleles, coefficients used in for the HLA-GRS calculation. Adapted from Moutsianas *et al*.,[5]

HLA allele or SNP	Parameter	OR	Beta Coefficients	Notation (β)	Allele Frequency in UKBB	In Geisinger	In FinnGen
HLA-DRB1*15:01	Additive Effect	3,92	1,37	β1	0.149	0.127	0.138
	Homozygote correction	0,54	-0,62	β1*HOM*			
HLA-A*02:01	Additive Effect	0,67	-0,4	β2	0.265	0.265	0.336
	Homozygote correction	1,26	0,23	β2*HOM*			
HLA-DRB1*03:01	Homozygote correction	2,58	0,95	β3*HOM*	0.144	0.098	0.101
HLA-DRB1*13:03	Additive Effect	2,62	0,96	β4	0.009	0.009	0.005
HLA-DRB1*08:01	Additive Effect	1,55	0,44	β5	0.018	0.033	0.094
rs9277565[T]	Additive Effect	1,32	0,28	β6	0.204	0.204	0.192
HLA-B*44:02	Additive Effect	0,78	-0,25	β7	0.106	0.082	0.062
HLA-B*38:01	Additive Effect	0,48	-0,73	β8	0.01	0.02	0.008
HLA-DQA1*01:01	Additive Effect in the presence of DRB1*15:01	0,65	-0,43	β9*INT*	0.142	0.138	0.185
rs2229092[C]	Additive Effect	1,33	0,29	β10	0.060	0.065	0.054

1.3 External validation: Geisinger, USA

1.3.1 Data Source and Population

We analysed data from 169,762 individuals in the Geisinger DiscovEHR cohort that has been described elsewhere.[6,7] Briefly, the Geisinger cohort is a cohort of individuals who sought healthcare at an outpatient and/or inpatient facility at Geisinger, an integrated health system in central and north-eastern Pennsylvania, USA. Individuals consented to participate in the MyCode Community Initiative to create a biorepository of blood, serum, and DNA samples for broad research use, including genomic analysis. MyCode samples are linked to Geisinger electronic health records (EHR). The study was conducted and reported according to the transparent reporting of a multivariable prediction model for individual prognosis or diagnosis (TRIPOD) guideline.[8] The Geisinger Institutional Review Board determined this study to be "Non-human subject research" using de-identified information (IRB #: 2023-1075).

1.3.2 Source of diagnosis based on diagnostic codes

Case and Control Identification

Data was extracted on 09 February, 2023. Cases were identified using ICD9/10 codes as detailed in **Supplementary Table 1**. Briefly, individuals with optic neuritis and/or multiple

sclerosis ICD9/10 codes were identified from encounter and problem list diagnoses from completed outpatient visits. Individuals with at least two codes for optic neuritis or multiple sclerosis more than 3 months apart in the Geisinger EHR are considered cases, those with one code were excluded, and those with no codes were considered controls.

Case Exclusion
Case exclusion criteria were similar to those used for the UKBB. Briefly, we removed cases with ICD9/10 codes for optic papillitis, nutritional optic neuropathy, and toxic optic neuropathy (**Supplementary Table 1**). In addition, individuals with only 1 encounter for codes in the case inclusion list (n=18) were also excluded. Due to individuals having multiple codes, the total number of individuals excluded by all criteria is 118.

1.3.3 Generating the MS-GRS

Genotyping
Samples were genotyped using Infinium OmniExpress Exome array (Illumina) and Infinium Global Screening Arrays (GSA-24v1.0 and GSA-24v2.0, Illumina). SNPs with minor allele frequency (MAF) ≤ 1%, significant deviation (p ≤ 1x10-15) from Hardy-Weinberg Equilibrium (HWE), and site-level missingness ≥ 1% were removed. Genotypes for each array were imputed to the TOPMED reference panel (97,256 deeply sequenced genomes) with a GRCh38 build using the TOPMED Imputation Server (https://imputation.biodatacatalyst.nhlbi.nih.gov), which employed Eagle v2.4 and Minimac4 as the phasing and imputation algorithm, respectively. When completed, the imputed data in VCF files were retrieved from the server and merged sample-wise using bcftools (https://samtools.github.io/bcftools/bcftools.html) in 5MB genomic regions. Only the imputed variants with info score ≥0.3 were considered for HLA imputation.

HLA imputation and haplotype formation
We used the HLA-TAPAS pipeline1[9] for imputation of HLA variants in 169,762 MyCode samples using a high-resolution HLA reference panel which captures global population diversity and enables multi-ancestry HLA imputation. The entire process included genotype data harmonization (alignment, deduplication, removing palindromic SNPs, sample and variant QC) at the MHC region, phasing (BEAGLE v4.1), and HLA imputation (SNP2HLA)[10] under a Linux environment. The entire MyCode sample was segmented with 5,000 randomly preselected samples per run in a parallel fashion. Finally, all the imputed data was aggregated. The MAF of the non-HLA variants at the MHC region were compared with those from UKB and FinnGen data (**Supplementary Data 1**) for internal consistency. HLA DQA1 and DQB1 genotype concordance for 130 MyCode samples from this imputation was compared to previously sequenced DQA1 and DQB1 alleles through a third-party diagnostic laboratory that used the SBT Resolver™ technology (CareDx, CA, USA). Results showed 99.23% concordance rates when compared to the 4th digit resolution.

GRS construction and estimation

MS-GRS was constructed using a combination of HLA and nonHLA variant scores. The effect size (β) of the risk alleles in the HLA haplotypes and nonHLA variants were estimated from IMSGC data.[3] MS-GRS score from the nonHLA variants was constructed by the default formula of so-called "sum score" (https://zzz.bwh.harvard.edu/plink/profile.shtml) of PLINK (version 1.9) using 306 (out of 307) mapped variants (info score >0.95) from the TOPMED imputed data without any missing genotypes. The inference of HLA haplotypes and the MS-GRS score formation for HLA variants was conducted by a customized procedure (https://github.com/ploginovic/MS-ON-ukb-code/). In the MS-free survival analysis, the final MS-GRS was z-score normalized to the undifferentiated ON group. Otherwise, non-normalised MS-GRS is reported to allow comparison between cohorts.

Covariates extraction from the Geisinger EHR

Only unrelated individuals up to third-degree relationship (n = 116,767) from the MyCode cohort were included in study. Relationships were inferred from cryptic relatedness analysis as previously described.[11] The index dates for control samples were calculated by subtraction of the last active date (primarily last encounter date) and birth date. The index ages for cases (MS or ON) were determined by the earliest date recorded for the ICD9/10 codes. Covariates included in the logistic regression model were reported sex, index age, and the first four principal components (PCs) (MS versus Control in Figure 2). When reported sex was unknown or missing, the genomic determined sex was used. Genomic determined sex, race (with or without EUR ancestry) and dichotomized index age for ON (18-50yrs) were used for the longitudinal study. Other risk factors were extracted from the Geisinger EHR without missing values (**Table 1**).

Data sharing

The HLA genotyping data and MS-GRS from the MyCode participants in this study may be shared with a third party for reasonable requests upon execution of the data-sharing agreement.

1.4 External validation: FinnGen, Finland

1.4.1 Data Source and Population

We conducted external validation using FinnGen data freeze 9 (R9) at individual level, including genome-wide genotype data and longitudinal healthcare registry data.[12,13] FinnGen (https://www.finngen.fi/en) is a public-private research project, combining genome and digital healthcare data since 2017. The FinnGen partners can be found at https://www.finngen.fi/en/partners. FinnGen R9 comprises 392,649 Finnish European individuals (n=173,010 men, n=219,496 women) with informed consent for biobank research based on the Finnish Biobank Act. Alternatively, separate research cohorts,

collected prior the Finnish Biobank Act came into effect (in September 2013) and start of FinnGen (August 2017), were collected based on study-specific consents and later transferred to the Finnish biobanks after approval by Fimea (Finnish Medicines Agency), the National Supervisory Authority for Welfare and Health. Recruitment protocols followed the biobank protocols approved by Fimea. The FinnGen study protocol (Nr HUS/990/2017) is approved by the Coordinating Ethics Committee of the Hospital District of Helsinki and Uusimaa (HUS). or regarding earlier cohorts, with approval from Fimea, the National Supervisory Authority for Welfare and Health. The FinnGen study protocol (number HUS/990/2017) is approved by the Coordinating Ethics Committee of the Hospital District of Helsinki and Uusimaa (HUS).

The FinnGen study is approved by Finnish Institute for Health and Welfare (permit numbers: THL/2031/6.02.00/2017, THL/1101/5.05.00/2017, THL/341/6.02.00/2018, THL/2222/6.02.00/2018, THL/283/6.02.00/2019, THL/1721/5.05.00/2019 and THL/1524/5.05.00/2020), Digital and population data service agency (permit numbers: VRK43431/2017-3, VRK/6909/2018-3, VRK/4415/2019-3), the Social Insurance Institution (permit numbers: KELA 58/522/2017, KELA 131/522/2018, KELA 70/522/2019, KELA 98/522/2019, KELA 134/522/2019, KELA 138/522/2019, KELA 2/522/2020, KELA 16/522/2020), Findata permit numbers THL/2364/14.02/2020, THL/4055/14.06.00/2020,,THL/3433/14.06.00/2020, THL/4432/14.06/2020, THL/5189/14.06/2020, THL/5894/14.06.00/2020, THL/6619/14.06.00/2020, THL/209/14.06.00/2021, THL/688/14.06.00/2021, THL/1284/14.06.00/2021, THL/1965/14.06.00/2021, THL/5546/14.02.00/2020, THL/2658/14.06.00/2021, THL/4235/14.06.00/202, Statistics Finland (permit numbers: TK-53-1041-17 and TK/143/07.03.00/2020 (earlier TK-53-90-20) TK/1735/07.03.00/2021, TK/3112/07.03.00/2021) and Finnish Registry for Kidney Diseases permission/extract from the meeting minutes on 4th July 2019.

The Biobank Access Decisions for FinnGen samples and data utilized in FinnGen Data Freeze 9 include: THL Biobank BB2017_55, BB2017_111, BB2018_19, BB_2018_34, BB_2018_67, BB2018_71, BB2019_7, BB2019_8, BB2019_26, BB2020_1, Finnish Red Cross Blood Service Biobank 7.12.2017, Helsinki Biobank HUS/359/2017, HUS/248/2020, Auria Biobank AB17-5154 and amendment #1 (August 17 2020), AB20-5926 and amendment #1 (April 23 2020) and it´s modification (Sep 22 2021), Biobank Borealis of Northern Finland_2017_1013, Biobank of Eastern Finland 1186/2018 and amendment 22 § /2020, Finnish Clinical Biobank Tampere MH0004 and amendments (21.02.2020 & 06.10.2020), Central Finland Biobank 1-2017, and Terveystalo Biobank STB 2018001 and amendment 25th Aug 2020.

1.4.2 Source of diagnosis based on diagnostic codes

In FinnGen, diseases are defined as endpoints by FinnGen clinical expert teams according to ICD codes of version 8 (1969-1986), 9 (1987-1995), and 10 (1996-2019).

In this analysis, ON endpoints include H7_OPTNEURITIS or H7_RETROBULBNEURINOTH and MS endpoint is G6_MS (**Supplementary Table 1**). ON cases were all the individuals with at least one ON diagnosis by the end of 2021. Individuals without any ON endpoint in the same time frame were defined as controls. More detailed information related to disease definitions and basic statistics can be found from https://risteys.finngen.fi/ by searching FinnGen endpoint.

1.4.3 Generating the MS-GRS

In FinnGen, the data was pre-processed by FinnGen analytical team considering HWE and data quality. After removing individuals with cryptic relatedness and individuals without clear demographic information (e.g., sex, age at DNA sample collection), our final study population consist of 372,416 individuals.

non-HLA-GRS

From the 307 non-HLA SNPs listed in **Supplementary Data 1**, 303 SNPs (out of 307) were successfully extracted (rs6441931, rs9366138, rs7923837, and rs11669861 were not available in FinnGen). The MAF of the extracted SNPs were consistent with those in UKBB and in Geisinger. We then used PLINK2 and the same weights to calculate GRS for the non-HLA regions.

HLA-GRS

The HLA alleles were imputed by a population-specific reference panel using FinnGen R9.[14] We adopted the same formula from Moutsianas *et al* and the same weights in **Supplementary Table 4** to calculate HLA-GRS.[5] For the HLA alleles, we performed scoring using Python 3 libraries Pandas and Numpy.[15,16] For the two SNPs in HLA regions, we used PLINK2 to score the genetic risk. After obtaining both HLA-GRS and non-HLA-GRS, we summed the two parts as MS-GRS. In the MS-free survival analysis, we normalized MS-GRS for undifferentiated ON group.

2. Supplementary results

2.1. External Validation Results in Geisinger (USA, PA) and FinnGen (Finland)

2.1.1. MS-GRS was discriminative of MS

Across all datasets, we calculated whether MS-GRS, and both HLA-GRS and non-HLA-GRS, differentiated between MS cases and healthy controls. **Supplementary Fig. 2** summarises he distribution of MS-GRS across datasets, and illustrates ROC-AUCs for MS-GRS, including its components, HLA-GRS and non-HLA-GRS.

In UKBB, both the HLA and non-HLA mean scores were higher in people with MS (mean 0.74 (SD 0.78) vs 0.29 (SD 0.71), $P < 0.0001$ for HLA, 2.88 (SD 0.92) vs 2.37 (0.92), $P < 0.0001$ for non-HLA) and were discriminative of MS (ROC-AUC (95% CI) 0.666 (0.663-0.669) and 0.656 (0.653-0.659) respectively) The full MS-GRS had a ROC AUC of 0.721 (0.718-0.723), and 0.752 (0.750-0.755) when combined with covariates.

In Geisinger, mean (95% CI) HLA scores were 0.503 (0.781) and 0.251 (0.687) for MS cases vs Controls (main Table 1), with both components being independently discriminative of MS: Mean ROC-AUCs (95% CI) of 0.602 (95% CI 0.599 - 0.605), and 0.615 (0.612 - 0.618) for HLA- and non-HLA-GRS respectively. MS-GRS had a ROC-AUC of 0.650 (0.648-0.653), compared to 0.721 (0.718-0.723) in UKBB. However, MS-GRS and covariates (reported sex, index age, and the first four principal components) performance were comparable between UKBB (Mean (95 %CI) 0.752, (0.750-0.755)) and Geisinger (0.744 (0.741-0.747)) due to high performance of the covariate (null) model (**Supplementary Fig. 2E**).

In FinnGen, both HLA and non-HLA performed better than in UKBB, with ROC-AUC for HLA-GRS of 0.664 (95% CI 0.661-0.667), and 0.682 (0.680-0.685) for non-HLA-GRS. Subsequently, final MS-GRS also performed better than in UKBB, with ROC-AUC of 0.737 (0.734-0.740) compared to UKBB's 0.721 (0.718-0.723). MS-GRS improved discrimination compared to covariates alone (sex, age at DNA sample collection, and the first four principal components), with ROC-AUC of 0.764 (95% CI 0.761-0.766) vs 0.647 (95% CI 0.644-0.650). Thus, the full model consisting of MS-GRS and covariates in FinnGen outperformed that of UKBB with ROC-AUCs of 0.764 vs 0.752, respectively. This could be explained by the fact that both non-HLA- and HLA-GRS were developed based on previously reported data by IMSGC without significant modification and adjustment to UKBB, thus avoiding overfitting in UKBB.

2.1.2 Genetic overlap of MS, ON and MS associated ON

In both Geisinger and FinnGen, the MS-GRS distribution pattern was generally similar to UKBB (**Figure 2**), with both MS-ON and MS Only cases having higher MS-GRS than healthy controls, and higher than ON only. One significant difference was observed in Geisinger where ON only did not differ significantly from healthy controls (Mean MS-GRS (SD) for ON only was 2.79 (1.21) vs 2.75 (1.13) in healthy controls, *P*=0.73).

Furthermore, in both Geisinger and FinnGen we observed that MS-GRS was significantly higher in MS-ON group compared to the rest of the MS cases. Specifically, in Geisinger, MS-GRS was 3.52 (1.22) in MS-ON, compared to 3.35 (1.31) in MS only (*P*-value < 0.0001), non-HLA-GRS for MS-ON was 2.96 (0.91) compared to 2.85 (0.98) in MS Only, and HLA-GRS was 0.56 (0.78) compared to 0.50 (0.78) in MS-ON vs MS only, respectively. In FinnGen, MS-GRS was 3.96 (1.21) in MS-ON, compared to 3.73 (1.25) in MS only (*P*-value < 0.0001), non-HLA-GRS for MS-ON was 3.15 (0.90) compared to 3.01 (0.93) in MS Only, and HLA-GRS was 0.81 (0.80) compared to 0.72 (0.78) in MS only.

Lastly, unlike UKBB and FinnGen, MS-GRS was significantly higher in Geisinger's subset of MS-ON cases where MS preceded ON (n=280) when compared to ON preceding MS (*P*<0.05, Welch's unequal variance t-test) **Supplementary Fig. 6.**

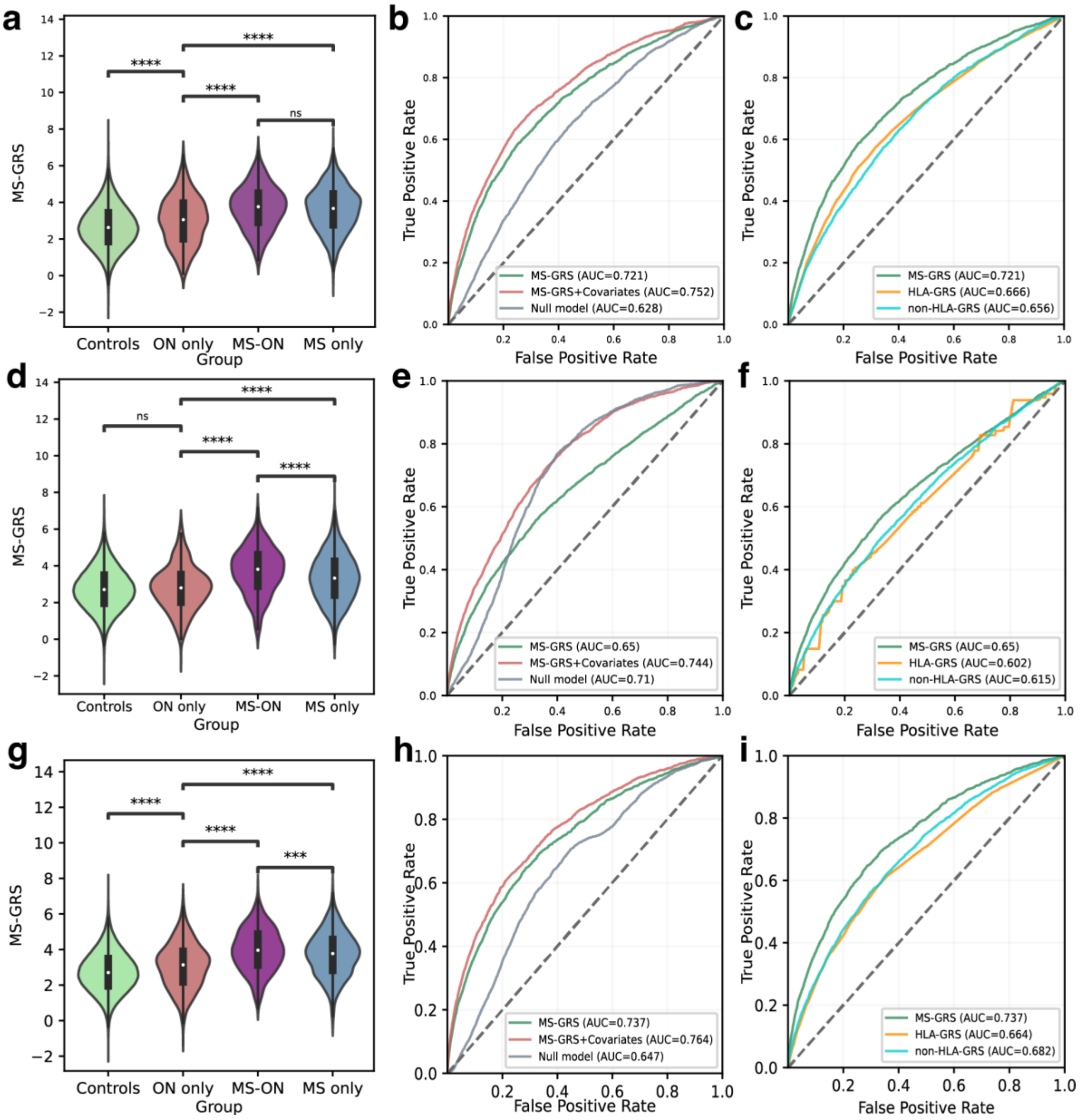

Supplementary Fig. 2. illustrates the distribution of the MS-GRS and its discriminative performance between all MS cases and healthy controls in our three datasets: UKBB (**A-C**), Geisinger (**D-F**), and FinnGen (**G-I**). Exact *P*-values are provided in Fig. 2 in the main manuscript.

2.1.3. Validating UKBB model of MS-free survival in undifferentiated ON

For the MS-free survival analysis in both Geisinger and FinnGen, we utilised the model trained on UKBB data. The model selected was the most-parsimonious model (discussed in main text and below), and consisted of MS-GRS, sex, and binary age at ON diagnosis (18 to 50 years of age). Weights (adjusted HR) of the final UKBB model are reported in Table 1. In both validation datasets, we utilized undifferentiated ON groups that were constructed by combining ON only with subset of MS-ON in who the diagnosis of ON preceded MS. In Geisinger, this group was comprised of 835 individuals, from which 140 (17%) subsequently

developed MS, at a median interval of 0.32 years (IQR 0.06–1.68) years from ON to MS diagnosis. In FinnGen, undifferentiated ON contained 977 patients, of which 369 (37.8%) developed MS until the end of follow-up, with the median (IQR) interval between ON and MS being one year (1.02, 0.24–5.46).

After adjusting the model predictions based on background risk of MS (cumulative hazard ratio at specific points in time) in undifferentiated ON group, the model calibrated well in both external validation datasets (**Supplementary Fig. 3 A-C**). In Geisinger, it was possible to achieve better calibration by accounting for the difference in duration of follow-up and censoring, but we aimed to keep the calibration curve adjustments consistent between datasets.

We then plotted observed Kaplan-Meier plots stratified into quartiles of predicted risk (predicted partial hazard). In both Geisinger and FinnGen, stratification by quartiles resulted in distinct observed risk quartiles (**Supplementary Fig. 3 D-F**). In Geisinger's low risk quartile, 6.7% (95% CI 3.7–10.1%) were diagnosed with MS at the end of the follow up, with the proportion of MS diagnosis being 9.6% (5.6–13.6%) in intermediate, 20.2% (14.7–25.6%) in higher, and 30.6% (24.4–36.9%) in the highest risk quartile. In FinnGen with the highest proportion of MS among undifferentiated ON of all cohorts, MS developed by the end of follow-up in 10.2% (95% CI 6.4-14.0%) in the lowest risk quartile, and 60.7% (54.5-66.8%) in the highest risk [36.1% (30.0-42.1%) and 42.2% (36.0-48.4% in the two middle quartiles].

Across all three datasets, the model containing MS-GRS performed better than covariates (Sex and binary age at ON diagnosis alone), as measured by time-dependant ROC-AUC (**Supplementary Fig. 4**).

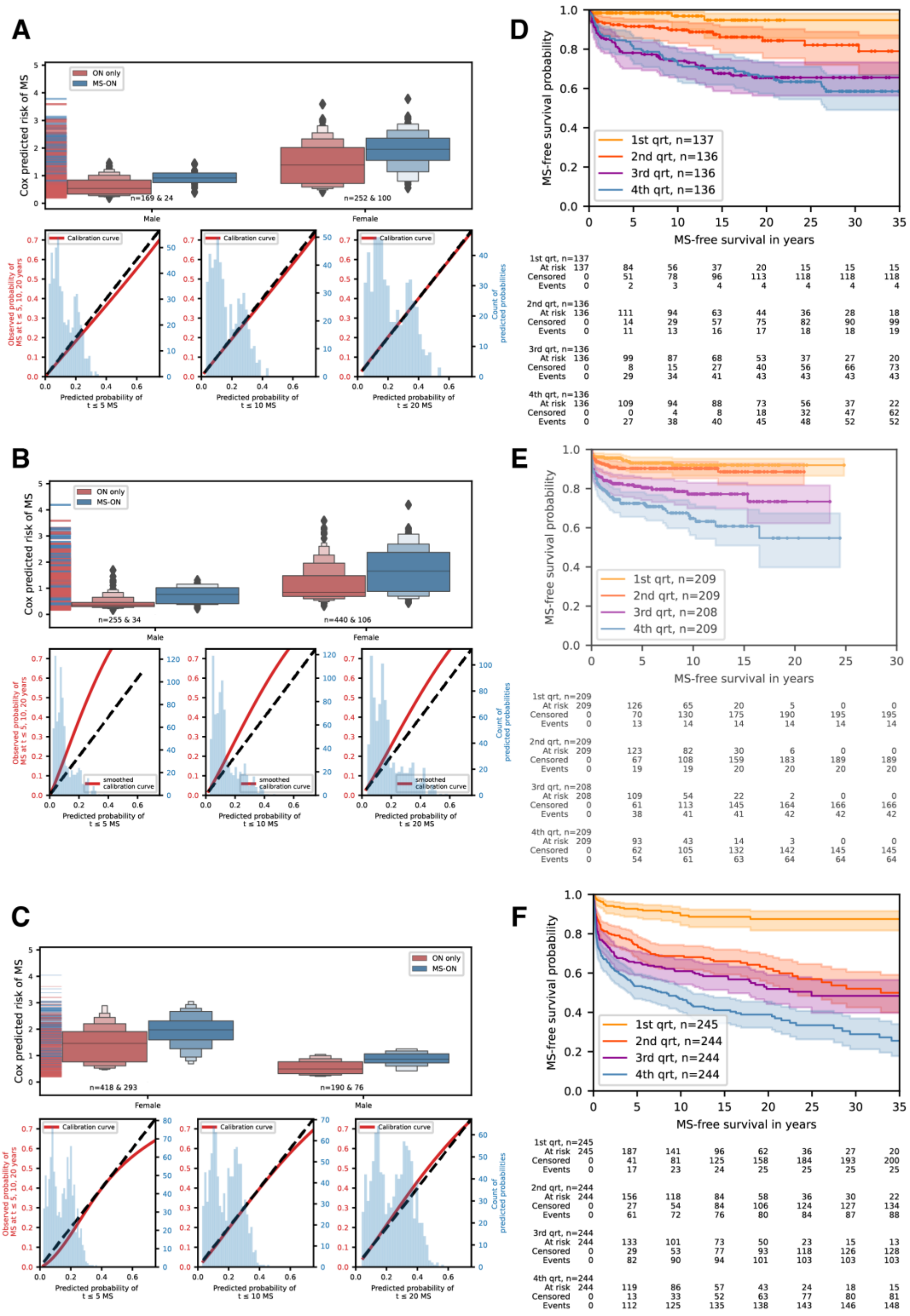

Supplementary Fig.3 A-C show the distributions of predicted partial hazard split by sex and whether or not MS was diagnosed by the end of cumulative follow-up (red boxes – no MS, blue – developed MS), and UKBB model calibration adjusted for MS frequency at three points in time. **A** is UKBB, **B** is Geisinger, **C** is FinnGen. Panels **D-F** demonstrate Kaplan-Meier plots of the MS-free survival in all three cohorts (**D-F** UKBB, Geisinger and FinnGen, respectively).

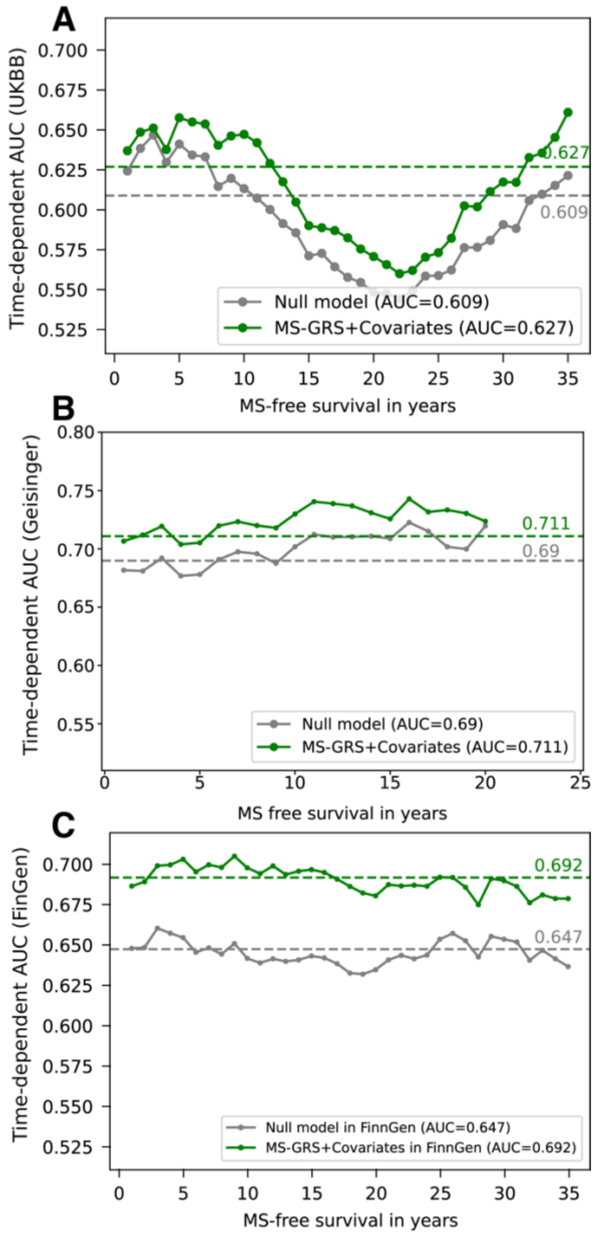

Supplementary Fig.4. A-C plots show time-dependant ROC-AUC for three datasets (UKBB, Geisinger, and FinnGen, respectively) comparing time-varying performance of the UKBB-trained cox models; full model in green (MS-GRS & covariates (Sex and binary age at ON diagnosis)) vs Null model (covariates alone). Dashed line shows the average time-ROC-AUC across all time-horizons. Note that **panel B** has different scales of both y- and x-axes due to higher ROC-AUC and shorter duration of follow-up, respectively.

2.2 UKBB Demographic data in all ON cases and all MS cases by end of cumulative follow-up

2.2.1. Additional UKBB-specific variables (Vitamin D, Country of Birth, Townsend Deprivation Index) & Tables for all ON and MS cases

Supplementary Table 5. **Additional variables not listed in Table 1 including variables specific to UK Biobank (UKBB).** *P*-values are derived from a from univariate MS-free Cox proportional hazard model in the undifferentiated ON UKBB population in the rightmost column.

Characteristic	Biobank	MS-ON at presentation	MS Only	Controls	Undifferentiated ON at presentation	Hazard Ratio of MS diagnosis amongst those with undifferentiated ON
**Ever smoked**						
No	**UKBB**	61 (43.0)	998 (47.5)	262,019 (54.5)	272 (49.9)	ref
	**Geisinger**	111(39.6)	761 (40.0)	53,094 (46.7)	377 (45.2)	
	**FinnGen**	97 (37.0)	579 (37.5)	164,907 (44.6)	382 (39.1)	
Yes	**UKBB**	80 (56.3)	1,085 (51.6)	216,213 (45.0)	267 (49.0)	0.89 (0.62-1.27), *P*=0.52
	**Geisinger**	169 (60.4)	1140 (60.0)	60,657 (53.3)	458 (54.9)	
	**FinnGen**	57 (21.8)	244 (15.8)	61,915 (16.8)	154 (15.8)	
Missing data	**UKBB**	1 (0.7)	20 (1.0)	2,458 (0.5)	6 (1.1)	0.74 (0.1-5.37), *P*=0.77
	**Geisinger**	0	0	0	0	
	**FinnGen**	108 (41.2)	721 (46.7)	142,811 (38.6)	441 (45.1)	
**Mean (SD) BMI, kg/m2**	**UKBB**	26.6 (5.2)	26.9 (5.0)	27.4 (4.8)	27.7 (5.1)	0.997 (0.83-1.2), *P*=0.97
	**Geisinger**	30.4(7.8)	29.7(7.4)	30.9(7.8)	31.1(7.8)	
	**FinnGen**	27.1 (6.7)	27.1 (5.9)	27.5 (5.5)	27.8 (6.1)	
Country of Birth	**UKBB**					
England		108 (76.1)	1,692 (80.5)	373,794 (77.8)	409 (75.1)	Ref
Wales		9 (6.3)	92 (4.4)	21,263 (4.4)	30 (5.5)	0.76 (0.28-2.08), *P*=0.60
Scotland		18 (12.7)	201 (9.6)	38,559 (8.0)	57 (10.5)	1.37 (0.82-2.27), *P*=0.23
Northern Ireland		0 (0)	12 (0.6)	296 (0.6)	4 (0.7)	1.06 (0.15-7.64), *P*=0.95
Republic of Ireland		0 (0)	13 (0.6)	4,733 (1.0)	7 (1.3)	0.6 (0.08-4.32), *P*=0.61
Elsewhere/not known		7 (4.9)	93 (4.4)	39,381 (8.2)	38 (7.0)	1.27 (0.64-2.53), *P*=0.49
Townsend Deprivation Index Quintile	**UKBB**					
1		47 (33.1)	733 (34.9)	165,737 (34.5)	184 (33.8)	ref
2		28 (19.7)	479 (22.8)	102,925 (21.4)	112 (20.6)	1.48 (0.93-2.35), *P*=0.10
3		26 (18.3)	311 (14.8)	79,731 (16.6)	94 (17.3)	1.30 (0.78-2.15), *P*=0.31
4		24 (16.9)	328 (15.6)	73,302 (15.3)	84 (15.4)	0.83 (0.45-1.5), *P*=0.53
5		17 (12.0)	249 (11.8)	58,411 (12.2)	71 (13.0)	0.90 (0.47-1.73), *P*=0.76

HLA-DRB1*15:01		Not included in Cox regression				
0 alleles	UKBB	61 (43.0)	1,091 (51.9)	356,077 (74.1)	354 (65.0)	
	Geisinger	159(56.8)	1,195(62.9)	87,487(76.9)	609(72.9)	
	FinnGen	117 (44.7)	804 (52.1)	275,311 (74.5)	614 (62.8)	
1 allele	UKBB	72 (50.7)	867 (41.2)	114,918 (23.9)	170 (31.2)	
	Geisinger	101(36.1)	640(33.7)	24,442 (21.5)	200(24.0)	
	FinnGen	116 (44.3)	633 (41.0)	87,118 (23.6)	320 (32.8)	
2 allele	UKBB	9 (6.3)	145 (6.9)	9,695 (2.0)	21 (3.9)	
	Geisinger	20(7.1)	66(3.5)	1,822(1.6)	26(3.1)	
	FinnGen	29 (11.1)	107 (6.9)	7,204 (1.9)	43 (4.4)	

Supplementary Table 6: Demographic data for all cases of ON, subdivided into MS and non-MS ON at end of 20.4 years (IQR 12.1–31.6) cumulative follow-up.

Characteristic	All MS-ON	ON without MS
Study participants (n)	266	421
Of which white (%)	224 (84.21)	359 (85.27)
Mean age at UKBB enrolment (SD, range)	54 (48-59, 40 to 69)	58 (51-63, 40 to 70)
n Females (F:M)	206 (3.43)	252 (1.49)
ON diagnosed between 18 and 50	197 (74.1)	231 (54.9)
Mean MS-GRS (SD)	3.71 (1.17)	3.02 (1.30)
**Ever smoked**		
No	124 (46.62)	209 (49.64)
Yes	140 (52.63)	207 (49.17)
Missing data	2 (0.75)	5 (1.19)
**Serum 25-hydroxyvitamin D level at baseline assessment**		
Sufficient (> 50 nmol/L)	88 (33.1)	139 (33.0)
Insufficient (25-50 nmol/L)	98 (36.8)	181 (43.00)
Deficient (<25 nmol/L)	57 (21.4)	65 (15.4)
Missing data	23 (8.7)	36 (8.6)
**Mean (SD) body mass index at UBKBB baseline assessment, kg/m2**	27.1 (5.3)	27.8 (5.0)
**Country of Birth**		
England	199 (74.81)	318 (75.53)
Wales	13 (4.89)	26 (6.18)
Scotland	36 (13.53)	39 (9.26)
Northern Ireland	1 (0.38)	3 (0.71)
Republic of Ireland	1 (0.38)	6 (1.43)
Elsewhere/not known	16 (6.02)	29 (6.89)
**Townsend Deprivation Index Quintile**		
1	85 (31.95)	146 (34.68)
2	62 (23.31)	78 (18.53)
3	51 (19.17)	69 (16.39)
4	39 (14.66)	69 (16.39)
5	29 (10.9)	59 (14.01)
Missing data	0 (0)	0 (0)
Died before end of follow-up (n)	9 (3.4)	13 (3.1)
Mean age at onset ON (SD, range)	42.73 (12.41, 16.8–73.5)	46.83 (15.55, 1.3-80.3)
Mean age at onset MS (SD, range)	41.84 (10.88, 18.4–	NA

	73.6)	
**HLA-DRB1*15:01**		
0	127 (47.74)	288 (68.41)
1 allele	121 (45.49)	121 (28.74)
2 allele	18 (6.77)	12 (2.85)

Supplementary Table 7: Demographic data for subgroups of patients all diagnosed with MS by end of cumulative follow-up.

**Characteristic**	**MS-ON at presentation**	**ON prior to MS**	**MS without ON**
Study participants (n)	142	124	2103
Of which white (%)	121 (85.2)	103 (83.06)	1,845 (87.7)
Mean age at UKBB enrolment (SD, range)	54.5 (48-59, 40 to 69)	53 (48-58, 40 to 69)	56 (49-62, 40 to 70)
n Females (F:M)	106 (2.9)	100 (4.2)	1,504 (2.5)
ON diagnosed between 18 and 50	89 (62.7)	108 (87.1)	NA
Mean MS-GRS (SD)	3.74 (1.15)	3.67 (1.19)	3.71 (1.17)
**Ever smoked**			
No	61 (43.0)	63 (50.8)	998 (47.5)
Yes	80 (56.3)	60 (48.4)	1,085 (51.6)
Missing data	1 (0.7)	1 (0.8)	20 (1.0)
**Serum 25-hydroxyvitamin D level at baseline assessment**			
Sufficient (> 50 nmol/L)	42 (29.6)	46 (37.1)	779 (37.0)
Insufficient (25-50 nmol/L)	52 (36.6)	46 (37.1)	771 (36.7)
Deficient (<25 nmol/L)	35 (24.7)	22 (17.7)	373 (17.7)
Missing data	13 (9.2)	10 (8.1)	180 (8.6)
**Mean (SD) body mass index at UBKBB baseline assessment, kg/m2**	26.6 (5.2)	27.6 (5.5)	26.9 (5.0)
**Country of Birth**			
England	108 (76.1)	91 (73.4)	1,692 (80.5)
Wales	9 (6.3)	4 (3.2)	92 (4.4)
Scotland	18 (12.7)	18 (14.5)	201 (9.6)
Northern Ireland	0 (0)	1 (0.8)	12 (0.6)
Republic of Ireland	0 (0)	1 (0.8)	13 (0.6)
Elsewhere/not known	7 (4.9)	9 (7.3)	93 (4.4)
**Townsend Deprivation Index Quintile**			
1	47 (33.1)	38 (30.7)	733 (34.9)
2	28 (19.7)	34 (27.4)	479 (22.8)
3	26 (18.3)	25 (20.2)	311 (14.8)
4	24 (16.9)	15 (12.1)	328 (15.6)
5	17 (12.0)	12 (9.7)	249 (11.8)
Missing data	0 (0)	0 (0)	3 (0.1)
Died before end of follow-up	6 (4.2)	3 (2.4)	149 (7.1)
Mean age at onset ON (SD, range)	47.3 (12.2, 20 to 73)	37.5 (10.4, 17-67)	NA
Mean age at onset MS (SD, range)	38.7 (9.6, 18 to 58)	45.5 (11.2, 21-74)	44.9 (12.5, 15 to 80)
**HLA-DRB1*15:01**			
0	61 (43.0)	66 (53.2)	1,091 (51.9)
1 allele	72 (50.7)	49 (39.5)	867 (41.2)
2 allele	9 (6.3)	9 (7.3)	145 (6.9)

2.2.2. Age at diagnosis of MS

For Multiple Sclerosis, we excluded one individual with a self-reported age at diagnosis of 0.5 years, as it was a clear outlier. 98% of the cases were between 19.5 and 73.75 years of age. The earliest report of MS was at 15.41 years, and the latest at 80.4 years. 5th and 95th percentiles were 24.67 and 67 years, respectively. Age at MS report is summarised in **Supplementary Fig. 5.**

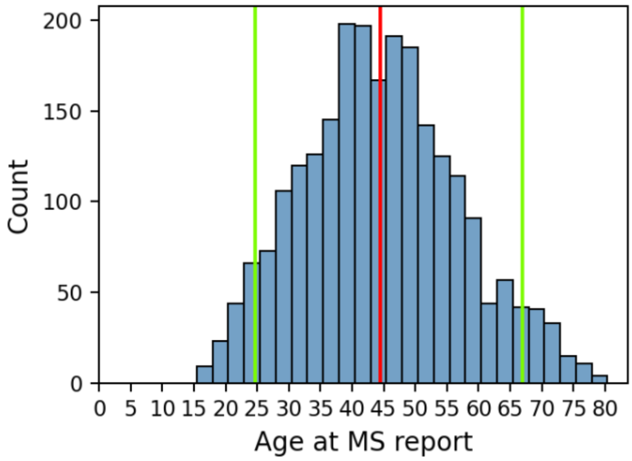

Supplementary Fig. 5. Histogram showing the distribution of age at MS diagnosis. The vertical red line shows the mean, green lines show the 5th and the 95th percentiles.

2.2.3 Age at diagnosis of ON

No exclusions were made based on age at ON diagnosis. Age at ON diagnosis ranged from 1 to 80 years, with the median (IQR) of 44.6 (35.1-55.4). 98% of cases were reported between 13.8 and 77.3 years of age, and 95th percentiles were 23.9 and 70.1 years, respectively. Age at ON report is summarised in **Supplementary Fig. 6.**

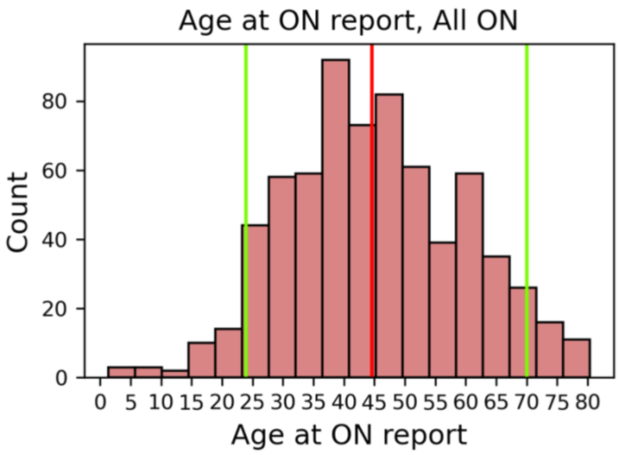

Supplementary Fig. 6. Age at ON report for All ON cases (n=687). The vertical red line shows the mean, green lines show the 5th and the 95th percentiles.

2.2.4 Age at diagnosis of ON in undifferentiated ON

In undifferentiated ON (n=545) 98% of the cases were between 9.8 and 78.2 years of age. The earliest report of MS was at 1.3 years, and the latest at 80.3. the 5th and 95th percentiles were 22.3 and 71.0 years, respectively. Age at ON report for undifferentiated ON is summarised in **Supplementary Fig. 7.** In ON that progressed to MS (n=124/545), 98% of ON were diagnosed between 17.3 and 63.3 years of age, with 5th and 95th percentiles being 22.8 and 53.8 years, respectively. 87.1% (109/124) of ON cases where ON progressed to MS were diagnosed with ON between 18 and 50 years of age. **Supplementary Fig. 8** summarises age at ON diagnosis in MS-ON vs ON only.

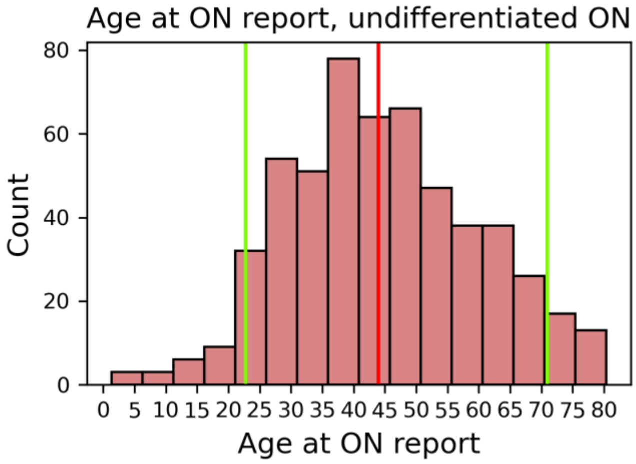

Supplementary Fig. 7. Histogram plot summarising age at ON diagnoses in undifferentiated ON (n=545). The vertical red line shows the mean, green lines show the 5th and the 95th percentiles.

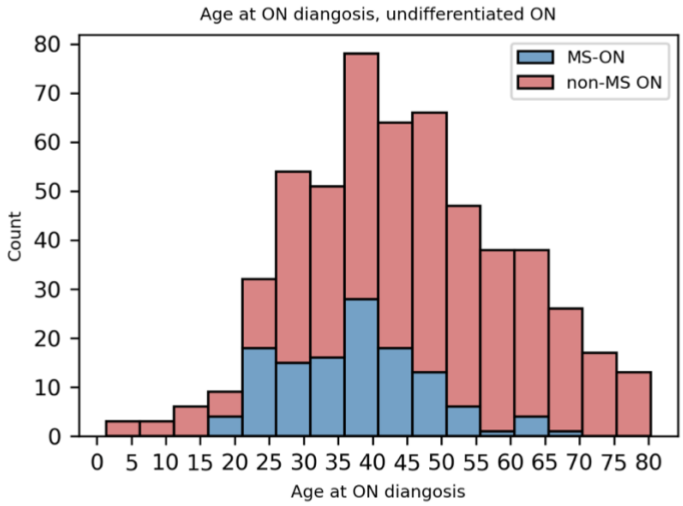

Supplementary Fig. 8. Histogram showing a stacked histogram summarising age at ON diagnosis in undifferentiated ON cases that progressed to MS (blue, n=124) versus those that did not (red, n=421). Red and blue bars are stacked on top of each other.

2.2.5 Causes of death in MS & ON

Causes of death are described below for undifferentiated ON & MS-ON at presentation in **Supplementary Table 8.**

Supplementary Table 8. Summarises causes of death for participants with undifferentiated ON (n=16) and MS-ON at presentation (n=6)

Group		Primary Cause of Death	Underlying cause of death	Underlying cause of death
**Undifferentiated ON at presentation**	**ON only**	I251 Atherosclerosis Heart	I259 Chronic ischaemic heart disease	
		E142 Diabetic Nephropathy	N185 stage 5 CKD	E149 Diabetes mellitus, unspec.
		K709 Alcoholic liver disease	K729 Hepatic coma	
		I679 Cerebrovascular disease, unspec.	R688 Abiotrophy	A419 Sepsis, unspec.
		C541 Endometrial cancer		
		C439 Malignant melanoma		
		C259 Pancreatic cancer	D70 Hyperleukocytosis	A419 Sepsis, unspec
		C160 Gastric cancer (cardia)	J189 Pneumonitis (Acute primary)	
		X815 Intentional self-half/ suicide	T07 Unspec multiple injuries	
		I609 Subarachnoid haemorrhage	G919 Macrohydrochephalus	I615 Bulbar haemorrhage
		F019 Vascular dementia	E119 Non-insulin-dependent DM	
		C80 Mesonephroma	D473 Essential thrombocytosis	
		C159 Oesophageal cancer		
	**Progressed to MS-ON**	G35 Multiple sclerosis	J189 Pneumonitis (Acute primary)	
		G35 Multiple sclerosis		
		G35 Multiple sclerosis	J180 Bronchopneumonia	
**MS-ON at presentation**		C64 Hypernephroma	G35 Multiple sclerosis	
		C349 Mal neoplasm of bronchus/lung, unspec	K315 Duodenal obstruction	K318 Apepsia
		J449 COPD, unspec	G35 Multiple sclerosis	
		A047 *C. difficile* enterocolitis	A419 sepsis, unspec	I10 Essential hypertension
		G35 Multiple sclerosis	J180 Bronchopneumonia	
		J440 COPD with acute LRTI	J22 Acute lower resp. infection, unspec	J449 COPD, unspec

2.3. First MS vs first ON in MS-ON

In UKBB, MS-GRS was not statistically different in people with MS in whom MS was reported before ON (MS first, n=122) when compared with those who ON progressed to MS (ON first, n=124). No significance was observed between the two groups in FinnGen. However, the difference was significant in Geisinger dataset **Supplementary Fig. 9B.**

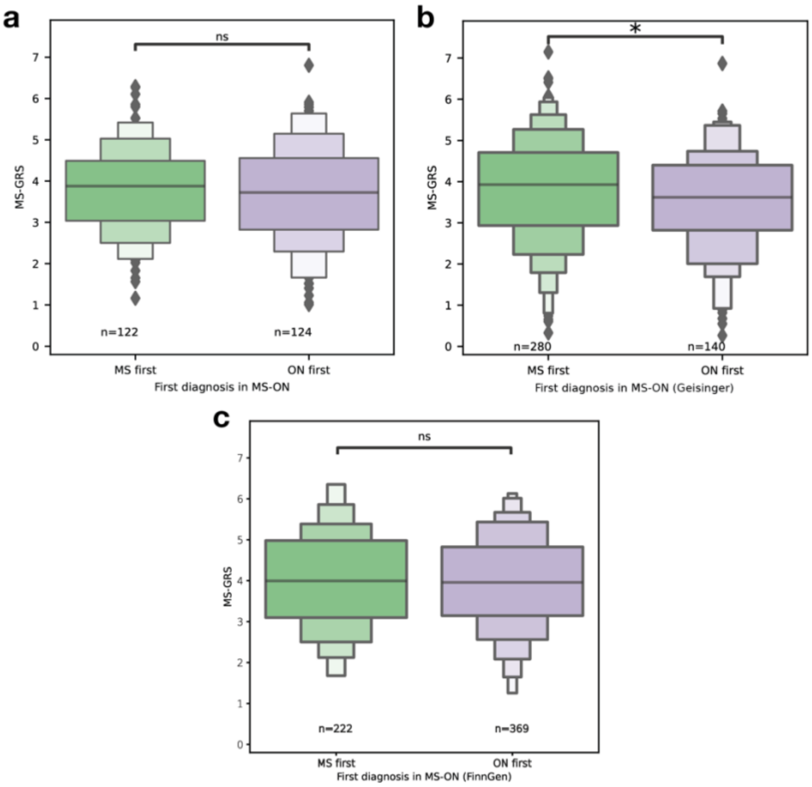

Supplementary Fig. 9 A-C box plots showing the distribution of MS-GRS in people with MS-ON in whom MS was diagnosed first vs ON diagnosed first. **A** is UKBB, **B** is Geisinger, **C** is FinnGen (*P*=; ns – not significant, * *P* < 0.05 (two-tailed Welch's *t*-test)

2.4. MS-GRS and other variables

2.4.1. Age at MS diagnosis

There was a weak (adjusted $R^2 = 0.0108$) yet significant ($P < 0.0001$) association between MS-GRS and Age at MS diagnosis, as shown in **Supplementary Fig. 10**

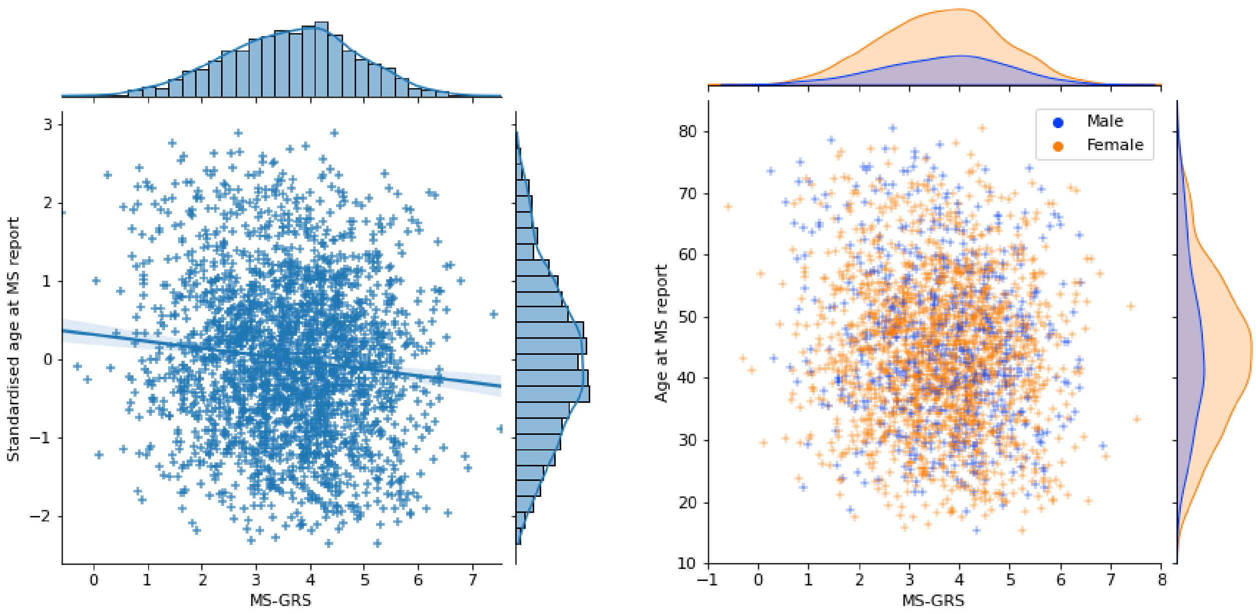

Supplementary Fig. 10 A. Scatterplot and regression showing a weak association between MS-GRS and age at MS diagnosis. **B.** Scatterplot showing the MS-GRS vs age at MS report in males and females**.**

2.4.2. Sex

There were no significant differences between the HLA-GRS in females and males in either group (controls, MS only, ON only, MS-ON), **Supplementary Fig. 11A**. non-HLA GRS was generally insignificantly different between cases and controls ($P = 0.067$, Unequal variance Welch's t-test), and significantly higher in men with MS only when compared to women with MS, $P < 0.05$, (**Supplementary Fig. 11B**).

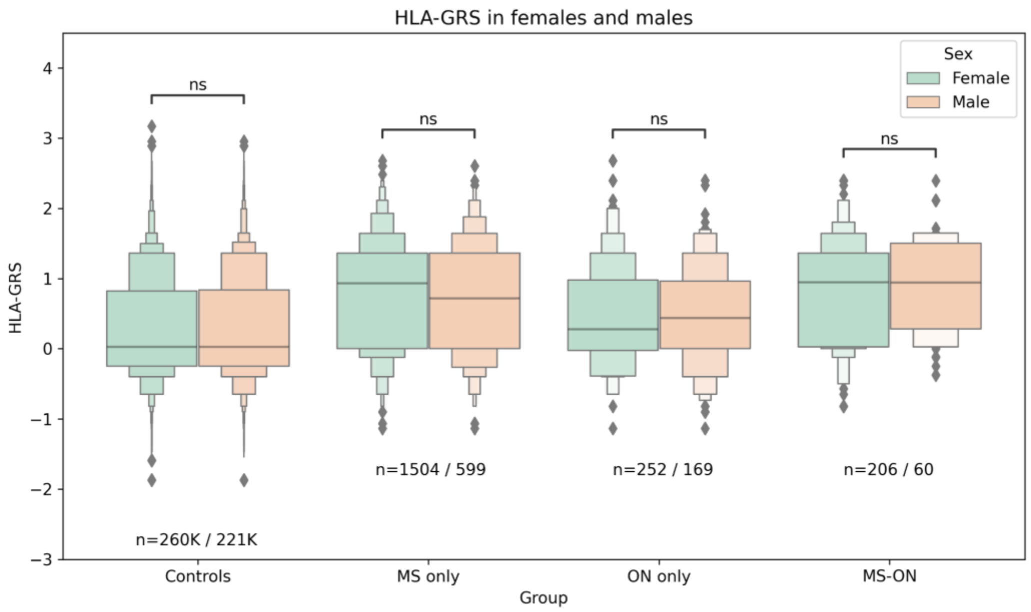

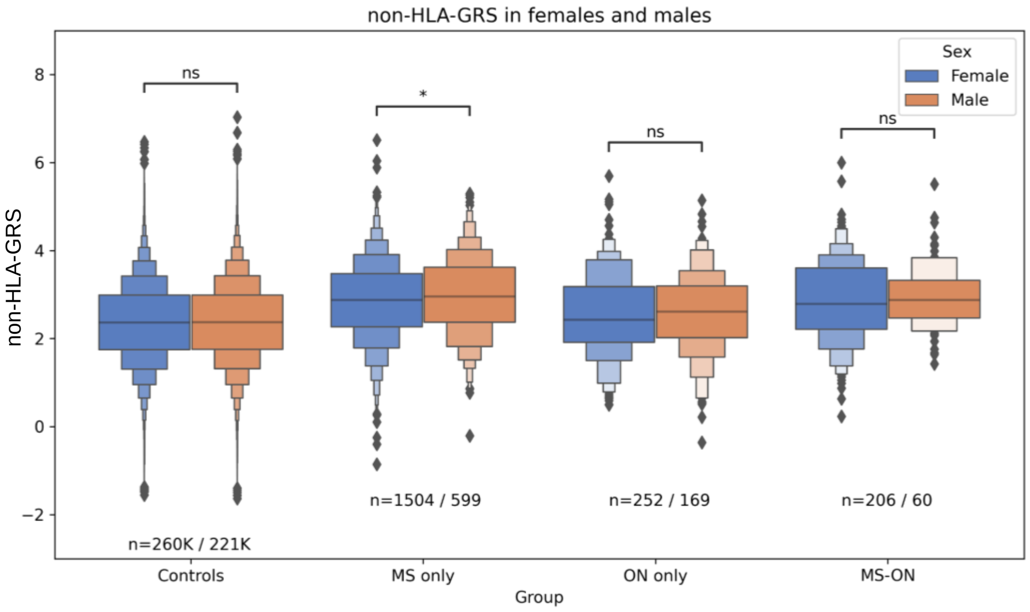

Supplementary Fig. 11. A. HLA-GRS distribution in Females and Males in four groups. **B.** non-HLA-GRS distribution in four groups. Welch's unequal variance t-test *P* value annotation: **ns:** $P > 0.05$; *: $0.01 < P < 0.05$

2.4.3. Ethnicity

We used the UKBB definition of ethnicity (Data-Field 22006) to visualise the difference of MS-GRS between ethnicities, although non-European populations are underrepresented in the UKBB. In most non-European populations, the number of MS cases was too low to allow meaningful statistical comparison (**Supplementary Fig. 12A**).

For the European ancestry British population used in sub-group analysis (see Section 3), we visualised MS-GRS in MS cases and controls stratified by binary ethnicity (**Supplementary**

Fig. 12B). MS-GRS did not differ significantly between MS cases irrespective of ethnicity (**Supplementary Fig. 12B**). European ancestry British controls had a slightly lower MS-GRS than non-European ancestry British controls (mean MS-GRS (SD), independent samples Welch's t-test with Bonferroni correction *P* value: 2.63 (1.16) vs 2.80 (1.14), $P < 0.0001$ for European ancestry British and the rest of UKBB, respectively), although the effect was small.

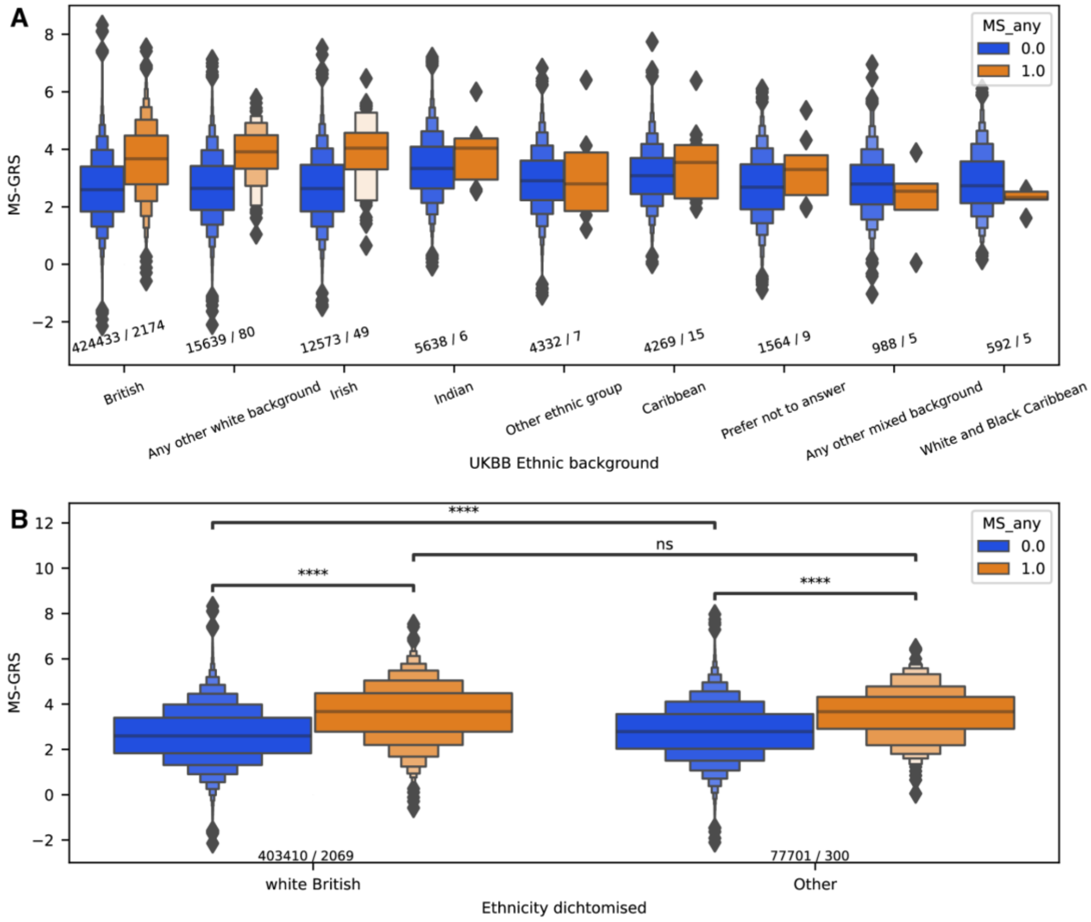

Supplementary Fig. 12. illustrates the MS-GRS difference between ethnicities. **A** uses ethnic groups defined by the UKBB. Numbers of MS cases and controls are shown under each pair of box plots. **B** shows MS-GRS by binary ethnicity and statistical significance of independent sample Welch's t-test with Bonferroni correction: ns $P > 0.05$; ****: $P < 0.0001$

2.5. Unadjusted Kaplan Meier of MS-free survival

We stratified the undifferentiated ON population (ON without baseline MS and did not die before the end of the follow-up) by quartiles of MS-GRS and plotted an empirical Kaplan-Meier MS-free survival plot (**Supplementary Fig. 13**).

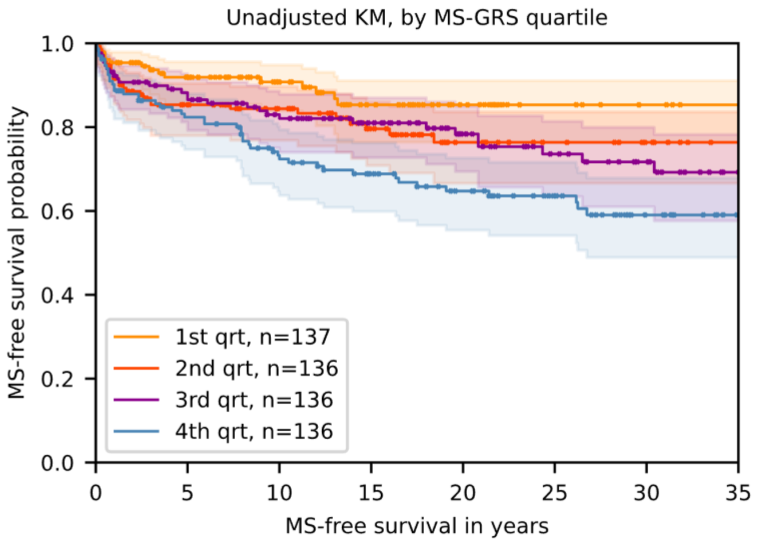

**1st qrt, n=137**								
At risk	137	98	75	53	40	27	22	19
Censored	0	29	51	69	82	95	100	103
Events	0	10	11	15	15	15	15	15
**2nd qrt, n=136**								
At risk	136	99	83	59	37	30	25	19
Censored	0	18	33	53	73	80	85	91
Events	0	19	20	24	26	26	26	26
**3rd qrt, n=136**								
At risk	136	103	89	73	54	41	31	18
Censored	0	16	25	40	57	67	76	88
Events	0	17	22	23	25	28	29	30
**4th qrt, n=136**								
At risk	136	103	84	71	59	46	29	19
Censored	0	10	17	26	34	46	60	70
Events	0	23	35	39	43	44	47	47

Supplementary Fig. 13. Unadjusted Kaplan Meier Plot Stratified by quartiles of MS-GRS showing MS-free survival for quartiles of MS-GRS

2.6. Cox Proportional Hazard Models

For the Cox survival regression models, we performed backward selection based on the partial Akaike Information Criterion (AIC). We then manually inspected *P* values of included covariates for statistical significance. For the initial set of covariates, we used variables reaching statistical significance ($P < 0.05$) in single variable analysis, biologically plausible covariates. Genetic principal components were not included to increase reproducibility across populations including Geisinger and FinnGen.

In the main MS-free survival model in undifferentiated ON, we used binary age at ON diagnosis and sex as covariates. However, adjusting for some other variables improved the model further, increasing the significance of MS-GRS in the model, and reducing the *P* value of a partial log-likelihood test comparing the resulting model to a null one (same covariates minus MS-GRS). We decided against including them in the main analysis, as the aim of our main findings was to propose a model that would be straightforward to replicate in a different dataset.

2.6.1. Final model proportional hazard assumptions

Proportional hazard assumptions of the final Cox model were assessed in UKBB to ensure the validity of our findings. Statistical analysis of the final MS-free cox survival model confirmed that the assumptions were not violated at *P*<0.05. Schoenfeld residuals, which provide a measure of proportional hazards assumption violations, were examined, and the graphical representation of Schoenfeld residuals are provided in **Supplementary Fig. 14** below.

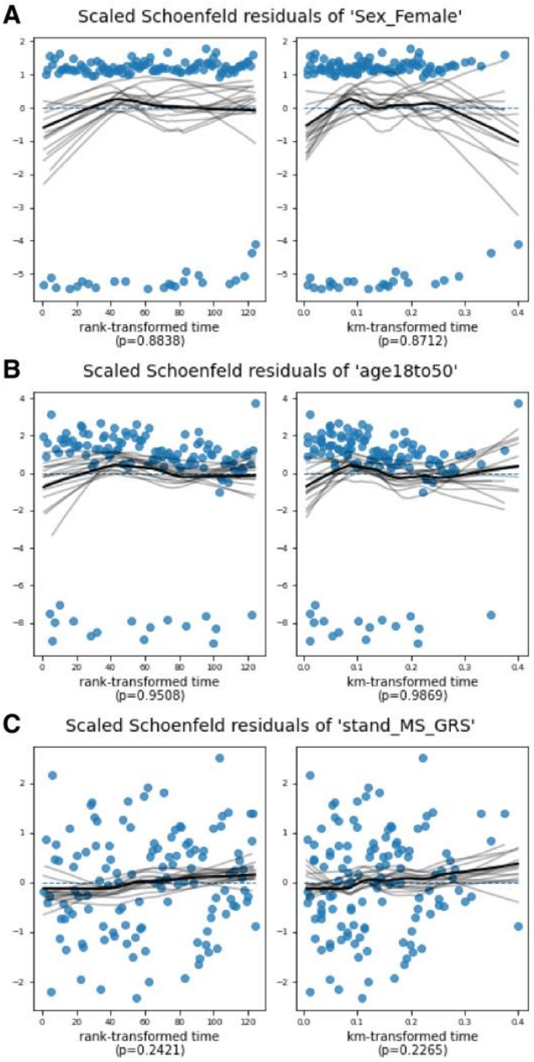

Supplementary Fig. 14 A-C Schoenfeld residuals of three variables of the final Cox proportional hazard model. Panel A is Sex (Female), B is binary age at diagnosis of ON [18-50], and C is MS-GRS standardised for undifferentiated ON population. Rank-transformed time on the left, and km-transformed time on the right of each panel, with *P*-values underneath each variable. Labels: Sex_Female – female sex, age18to50 – binary age at ON diagnosis from 18 to 50 years of age, stand_MS_GRS - standardised MS-GRS.

2.6.2. Final model Hazards for quartiles and composition of quartiles

We looked at how the final model's predicted partial hazard for MS was distributed within each quartile of predicted risk, and the sex composition of each quartile. The results are summarised below in **Supplementary Fig. 15** and **Supplementary Fig. 16**.

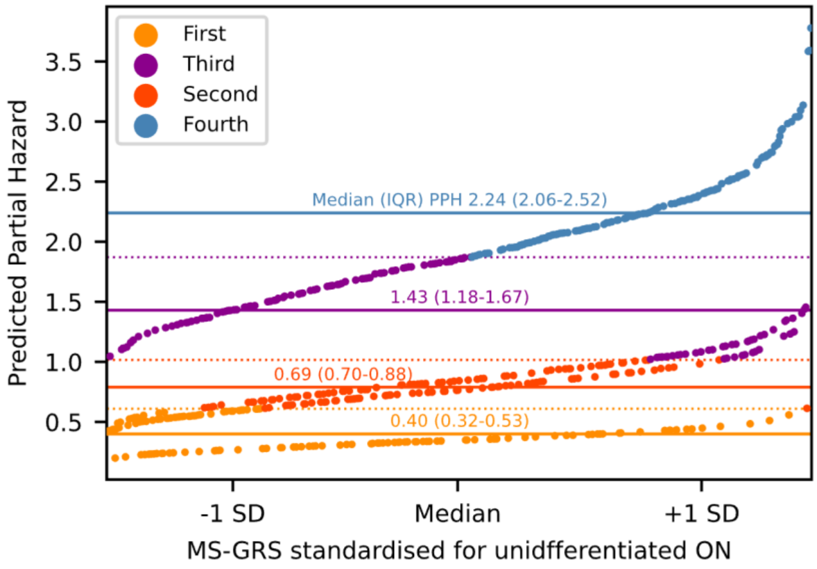

Supplementary Fig. 15. Plot illustrating how the distribution of MS-GRS affected the quartiles and displaying the median (IQR) predicted partial hazard (PPH) for each quartile as solid lines. Dotted lines indicate the cut-off point in predicted partial hazard between two quartiles. Note the four strata, which are (from bottom to the top): men with ON diagnosis outside of 18 to 50 y.o., women with ON diagnosis outside of 18 to 50, men diagnosed with ON aged 18-50, and women diagnosed with ON aged 18 to 50.

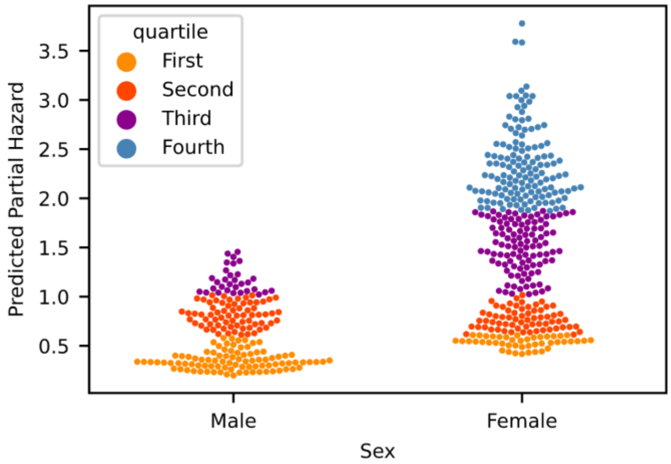

Supplementary Fig. 16. Swarmplot demonstrating the composition of each quartile of predicted partial hazard (PPH) in UKBB. Female: male ratio within each quartile is shown in main text Fig. 5d.

2.6.3. Continuous age at ON diagnosis modelling

We explored whether using continuous age at ON diagnosis performed better in MS-free survival cox model than a binary variable of age at ON diagnosis which captured ON diagnosis between 18 and 50 years of age and was selected based on clinical observations and epidemiological studies, which were mirrored in UKBB. We found that including continuous age at ON diagnosis in UKBB model deemed age at ON not significant in the model containing standardised MS-GRS and Sex (*P*-value = 0.16) and inflated the effect size of MS-GRS and sex (**Supplementary Table 9**). Furthermore, overall, the model with continuous age at ON diagnosis performed significantly worse compared to binary age at ON diagnosis [18-50], both based on models partial Akaike's Information Criterion (AIC) (1405.39 vs 1415.73, for binary vs continuous age at ON), and partial log-likelihood (-699.7 vs -704.9, respectively, log-likelihood ratio test *P*-value = 0.00131 on 1 degree of freedom). The table below (Supplementary Table 9) summarises both models, and **Supplementary Fig. 17** shows that no assumptions of proportional hazard were violated at *P*<0.05.

Supplementary Table 9: Summary of the three competing models: MS-GRS, sex, and binary age at diagnosis (top), MS-GRS, sex and continuous age at ON diagnosis (middle), MS-GRS, sex, binary age at ON diagnosis and an interaction term between sex and binary age at ON (bottom). aHR – adjusted Hazard Ratio, aHR lower/upper 95% – boundaries of 95% confidence interval, partial AIC – partial Akaike's Information Criterion. *P*-values derived from a chi-squared test for each variable within a respective Cox proportional hazard model.

	model	Final (MS-GRS, Binary age at ON diagnosis [18-50], Sex)				
**Variable**						
	**aHR**	**se(aHR)**	**aHR lower 95%**	**aHR upper 95%**	***P*-value**	**-log2(*P*)**
**MS-GRS**	1.29	0.09	1.07	1.55	0.01	7.22
**Sex_Female**	2.2	0.23	1.41	3.45	<0.005	10.87
**age18to50**	2.43	0.28	1.41	4.17	<0.005	9.53
**Partial AIC**	1405.39		**partial log-likelihood**	-699.7		

	model	Continuous age at ON onset instead of Binary age at ON diagnosis				
**Variable**						
	**aHR**	**se(aHR)**	**aHR lower 95%**	**aHR upper 95%**	***P*-value**	**-log2(*P*)**
**MS-GRS**	1.38	0.09	1.15	1.65	<0.005	11
**Sex_Female**	2.3	0.23	1.47	3.59	<0.005	11.9
**Cont. age ON**	0.99	0.01	0.98	1	0.16	2.65
**Partial AIC**	1415.73		**partial log-likelihood**	-704.86		

	model	Final + Interaction between Binary age at ON and Sex				
**Variable**						
	**aHR**	**se(aHR)**	**aHR lower 95%**	**aHR upper 95%**	***P*-value**	**-log2(*P*)**

**stand_MS_GRS**	1.3	0.09	1.08	1.57	0.01	7.54
**age18to50**	5.37	0.74	1.26	22.91	0.02	5.43
**Sex_Female**	5.2	0.76	1.18	22.94	0.03	5.09
**Interaction: age18to50*Sex_F**	0.38	0.79	0.08	1.78	0.22	2.2
**Partial AIC**	1405.56		**partial log-likelihood**	-698.9		

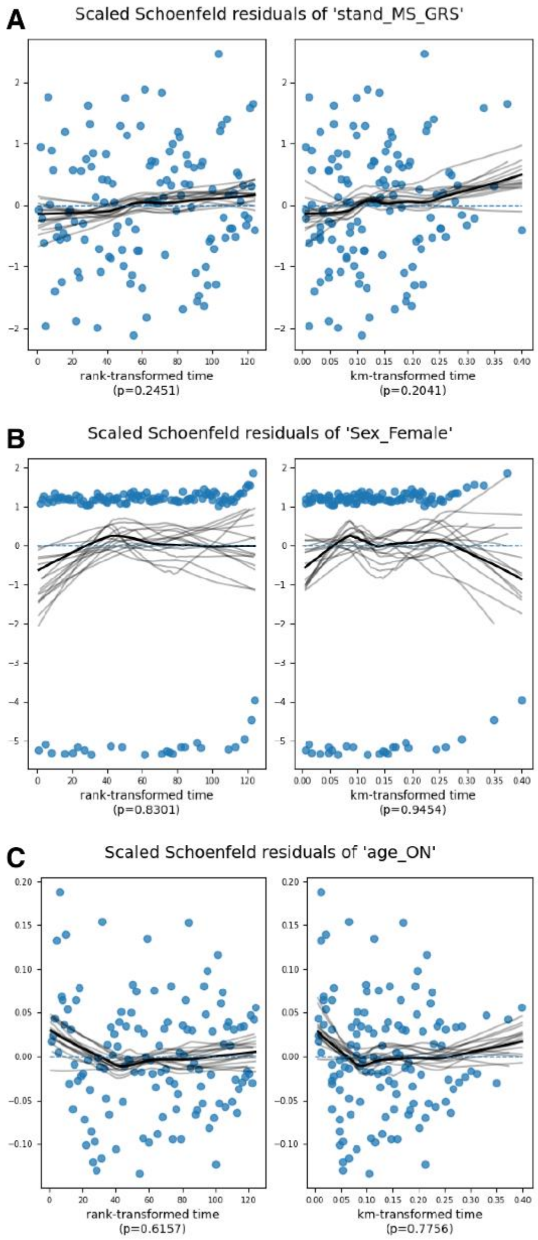

Supplementary Fig. 17. Scaled Schoenfeld residuals for a model with continuous age at ON diagnosis. Panel A is Sex (Female), B is continuous age at ON diagnosis, and C is MS-GRS standardised for undifferentiated ON population. Rank-transformed time on the left, and km-transformed time on the right of each panel, with *P*-values underneath each variable. stand_MS_GRS - standardised MS-GRS, Sex_Female – female sex, age_ON – continuous age at ON diagnosis in years

2.6.4. Interaction Sex*Age at ON diagnosis

We explored whether including an interaction term between female sex and binary age at age of ON diagnosis improved the model. However, the interaction term was not significant in the model (*P*= 0.217), and despite marginal improvements in partial AIC and partial log-likelihood (See **Supplementary Table 9**), the partial log-likelihood ratio test comparing a model with vs without an interaction term was not significant (*P*-value = 0.176, 1 DF), and thus the interaction term was not included.

2.6.5. Adjusting for Age at UKBB enrolment

Age at UKBB enrolment was significant in the model with MS-GRS, binary age at ON and sex (*P* value < 0.005, adjusted HR (95% CI) 0.94 (0.92-0.97)), with younger entrants of UKBB being more likely to be diagnosed with MS-ON rather than ON (**Supplementary Table 9**). Adjusting for age at UKBB entry increased the significance of MS-GRS in the model and resulted in better overall prediction of future MS (adjusted HR (95% CI) 1.36 (1.13-1.63), *P* = 0.00103) (**Supplementary Fig. 18**). **Supplementary Table 10** compare the final model with and without the addition of age at UKBB entry. we did not include age at UKBB entry into the model to allow external validation, as age at entry to other cohorts may have been different. Furthermore, the **Supplementary Fig. 18** shows better stratification when the model was adjusted for cohort entry, with a greater effect of MS-GRS. Lastly, we did not calculate time-ROC-AUCs for this model. While it is important to acknowledge the bias, it is specific to UKBB and is unlikely to be replicated elsewhere.

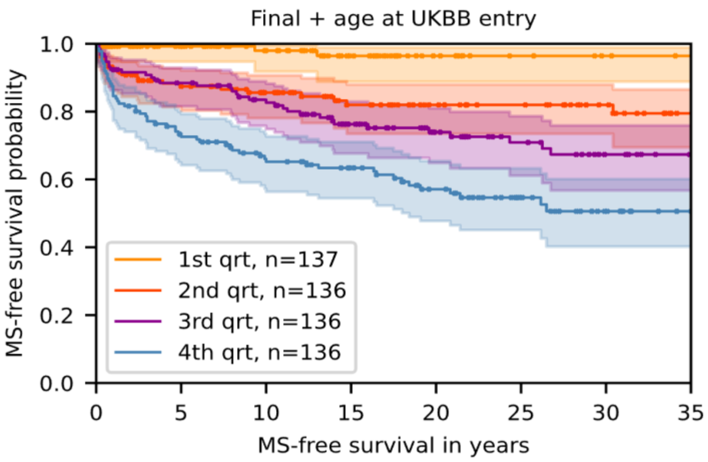

	0	5	10	15	20	25	30	35
**1st qrt, n=137**								
At risk	137	97	74	53	34	27	24	22
Censored	0	39	61	81	100	107	110	112
Events	0	1	2	3	3	3	3	3
**2nd qrt, n=136**								
At risk	136	101	84	64	51	44	34	21
Censored	0	19	34	51	64	71	81	93
Events	0	16	18	21	21	21	21	22
**3rd qrt, n=136**								
At risk	136	111	97	73	56	42	32	25
Censored	0	10	17	34	49	61	69	76
Events	0	15	22	29	31	33	35	35
**4th qrt, n=136**								
At risk	136	94	76	66	49	31	17	7
Censored	0	5	14	22	33	49	61	71
Events	0	37	46	48	54	56	58	58

Supplementary Fig. 18. Kaplan-Meier of undifferentiated ON MS-free survival plotted by quartile of MS risk. Quartiles were defined by quartiles of predicted partial hazard of MS by the Cox model.

Supplementary Table 10. Summary of two models for UKBB cohort: the final model with MS-GRS, binary age at ON diagnosis [18-50] and Sex (top), vs Final model adjusted for age at UKBB entry. aHR–adjusted Hazard Ratio, aHR lower/upper 95% – boundaries of 95% confidence interval, partial AIC – partial Akaike's Information Criterion. *P*-values derived from a chi-squared test for each variable within a respective Cox model.

	**model**	Final (MS-GRS, Binary age at ON diagnosis [18-50], Sex)				
**Variable**						
	**aHR**	**se(aHR)**	**aHR lower 95%**	**aHR upper 95%**	***P*-value**	**log2(*P*)**
**MS-GRS**	1.29	0.09	1.07	1.55	0.01	7.22
**Sex_Female**	2.2	0.23	1.41	3.45	<0.005	10.87
**age18to50**	2.43	0.28	1.41	4.17	<0.005	9.53
**Partial AIC**	1405.39		**partial log-likelihood**	-699.7		
	**model**	Final + Age at UKBB entry				
**Variable**						
	**aHR**	**se(aHR)**	**aHR lower 95%**	**aHR upper 95%**	***P*-value**	**-log2(*P*)**
**MS-GRS**	1.36	0.09	1.13	1.63	<0.005	9.92
**Sex_Female**	2.22	0.23	1.42	3.48	<0.005	11.1
**Age at UKBB entry**	0.94	0.01	0.92	0.97	<0.005	17.93
**age18to50**	1.73	0.29	0.98	3.03	0.06	4.12
**Partial AIC**	1385.76		**partial log-likelihood**	-688.9		

3. Supplementary and Sensitivity Analyses

This part described the primary analysis approach limited to the white unrelated European ancestry population only, who form the largest racial/ethnic subgroup in the UKBB dataset. Furthermore, we provide sensitivity analyses on non-white European population

3.1. White European Population definition

The white European population was defined based on the UKBB data-field 22006, which defines European ancestry based on self-reported data and genetic principal components (See https://biobank.ndph.ox.ac.uk/ukb/field.cgi?id=22006).

3.1.1. Cases and demographic characteristics

From 405,479 unrelated individuals of white European origin with available genetic and phenotype data, we identified 1845 MS cases, the vast majority of whom came from at least two sources. We identified 583 ON cases diagnosis, including 172 cases with diagnostic Read3 codes (F4H3 or F4H32) omitted from the UKBB definition of ON. Of these, 224 (38.4%) were diagnosed with MS during cumulative follow-up. This was similar to 39% MS-ON in our primary analysis.

We analysed demographic characteristics and present results in **Supplementary Table 8** below. Female: male ratio remained similar in white European population in all groups: MS-ON (3.39), MS only (2.54), ON only (1.39).

We found that from 224 people with MS-ON, 103 people had ON diagnosed first, 105 had MS diagnosed first, and 16 people had both diagnoses appear on the same day.
For the MS-free survival analysis, we limited our primary analysis to 'undifferentiated' ON cases after excluding people with MS diagnosed before ON (n=105 out of 224 MS-ON cases), simultaneous MS-ON cases (n=16 out of 224).

Supplementary Table 11. Demographic characteristics of white British population. *P*-values in the rightmost column are derived from a from univariate MS-free Cox proportional hazard model in the undifferentiated ON UKBB population, unless specified otherwise with an asterix: * *P*-values from multivariate Cox MS-free survival model with binary age at ON diagnosis, sex, and MS-GRS; ** *P*-values from a model with binary age at ON diagnosis, sex, MS-GRS and age at UK Biobank (UKBB) enrolment.

Characteristic	MS-ON at presentation	MS Only	Controls	Undifferentiated ON at presentation	Adjusted Hazard Ratio of MS diagnosis amongst those with undifferentiated ON
Study participants (n)	121	1,845 (87.73)	403,051 (83.85)	462	
Median age at UKBB enrolment (IQR, range)	55 (49-59, 41-69)	54 (48.5-59, 41-69)	58 (51-63, 41-69)	58 (50-63, 41-69)	0.995 (0.9924-0.997), *P*<0.0001**
**n Females (F:M)**	91 (3,03)	1,322 (2,53)	217,370 (1.17)	291 (1,7)	**2.18 (1.34-3.52), *P*=0016***
**ON diagnosed between 18 and 50**	75 (61.98)	NA	NA	288 (62.34)	**2.52 (1.52-4.58), *P*=0.0028***
**Mean MS-GRS (SD)**	3.81 (1.10)	3.62 (1.23)	2.63 (1.16)	3.19 (1.32)	**1.29 (1.07–1.55), *P*=0.0158***
**Ever smoked**					
No	54 (44.63)	892 (48.35)	218,897 (54.31)	225 (48.7)	–
Yes	66 (54.55)	937 (50.79)	182,626 (45.31)	234 (50.65)	1.04 (0.7-1.53), *P*=0.86
Missing data	1 (0.83)	16 (0.87)	1,528 (0.38)	3 (0.65)	1.38 (0.19-10.04), *P*=0.75
**Serum 25-hydroxyvitamin D level at baseline assessment**					
Sufficient (> 50 nmol/L)	38 (31.40)	695 (37.67)	173,306 (43.00)	157 (33.98)	–
Insufficient (25-50 nmol/L)	43 (35.54)	677 (36.69)	152,445 (37.82)	197 (42.64)	0.78 (0.49-1.23), *P*=0.28
Deficient (<25 nmol/L)	29 (23.97)	316 (17.13)	44,104 (10.94)	69 (14.94)	1.27 (0.73-2.2), *P*=0.39
Missing data	11 (9.09)	157 (8.51)	33,196 (8.24)	39 (8.44)	0.97 (0.47-2.02), *P*=0.94
**Mean (SD) body mass index at UBKBB baseline assessment, kg/m2**	26.9 (5.4)	26.9 (5.0)	27.4 (4.8)	27.7 (5.0)	0.99 (0.95 - 1.03), *P*=0.69
**Country of Birth**					–
England	96 (79.34)	1,542 (83.58)	340,673 (84.52)	371 (80.30)	0.73 (0.23-2.32), *P*=0.59
Wales	7 (5.79)	85 (4.61)	19,658 (4.88)	28 (6.06)	0.79 (0.29-2.17), *P*=0.65
Scotland	18 (14.88)	179 (9.70)	34,209 (8.49)	49 (10.61)	1.38 (0.79-2.4), *P*=0.20
Northern Ireland	/	9 (0.49)	1,864 (0.46)	4 (0.87)	1.13 (0.16-8.1), *P*=0.91
Republic of Ireland	/	/	225 (0.06)	/	NA
Elsewhere/not known	/	30 (1.63)	6,422 (1.59)	10 (2.16)	1.37 (0.43-4.34), *P*=0.59
**Variables not included in Cox regression analysis**					
Died before end of follow-up (n)	6 (5.0)	129 (7.0)	16,442 (4.1)	13 (2.8)	
Mean age at onset ON (Sd, range)	43.2 (11.9, 19.9-73.5)	NA	NA	44.7 (15.1, 1.3-80.3)	
Mean age at onset MS (SD, range)	38.4 (9.82, 18–58)	44.9 (12.5, 15.4-80.4)	NA	45.8 (10.9, 20.5-73.6)	
**HLA-DRB1*15:01**					
0	50 (41.32)	937 (50.79)	294,979 (73.19)	297 (64.29)	
1 allele	63 (52.07)	775 (42.01)	99,577 (24.71)	147 (31.82)	
2 allele	8 (6.61)	133 (7.21)	8,495 (2.11)	18 (3.90)	

3.1.2. MS-GRS ROC-AUC

We assessed the ability of MS-GRS to discriminate between all MS cases and healthy controls in white Europeans only in UKBB using ROC-AUC (**Supplementary Fig. 19A)**. Both the HLA and non-HLA scores were independently discriminative of MS (ROC-AUC 0.668 (95% CI 0.665-0.671) and 0.663 (0.660-0.666) respectively, **Supplementary Fig. 19B**) and had a combined ROC AUC of 0.726 (0.723-0.729). This was only a slight improvement when compared to the performance of MS-GRS on the whole of the UKBB (0.721 (0.718-0.723). When combined with a subset of known risk factors for MS, namely sex, age at UKBB entry, Townsend deprivation index and first four PCs, the ROC-AUC was 0.749 (0.747 - 0.752) (compared to 0.752 the whole of UKBB). This could be explained by some covariates, such as genetic principal components, not being as significant in the white only population.

3.1.3. Genetic overlap of MS, ON and MS associated ON

We first assessed the distribution of MS-GRS in Controls, ON only, MS-ON and MS only (**Supplementary Fig. 20 A,B**). The pattern was the same in White European only as in the whole of the UKBB, with the same significant differences.

3.1.4. MS-GRS predictive of future MS in individuals with ON

We assessed MS-GRS in 462 individuals with a first occurrence of ON where either there was no pre-existing diagnosis of MS or there was no diagnosis of MS at the same time (**Supplementary Table 11**). This included 373 individuals with ON who never received a diagnosis of MS with a median of 17.8 (IQR 9.5-30.1) years of follow up. Both prevalent (before UKBB entry) and incident (after UKBB entry) were used, with 89/462 of ON diagnoses being incident. 103 individuals had an ON diagnosis that was followed by a diagnosis of MS, with a median interval of 3.42 years (IQR 0.71–9.60) years.

When the Cox hazard model was fitted with MS-GRS, Sex and binary age at diagnosis, MS-GRS was significant in the model ($P < 0.01$); the Hazard Ratio (95% CI) of future MS diagnosis amongst participants presenting with undifferentiated ON was 1.29 (1.05-1.58, $P < 0.05$) per standard deviation increase in MS-GRS. Stratifying by the predicted risk quartiles, each subsequent quartile had a higher occurrence of MS-ON, as shown in **Supplementary Fig. 22.** This identified people who were at relatively low risk of MS (Percent diagnosed with MS-ON at the end of the follow up, 95% CI: 3.4% (0.1%–6.8%), intermediate risk groups 13.9% (7.6% –20.2%), higher MS risk group, 31.3% (22.8%–39.8%) and highest MS risk group 40.5% (31.6%–49.5%) (**Supplementary Fig. 23**)**.** Importantly, the model with MS-GRS performed significantly better than the null model with covariates only (Partial log-likelihood ratio test P value < 0.01 on 1 DF). No variables violated the assumptions of proportional hazard were violated at $P < 0.05$ in this model (not shown).

It is worth noting that adjusting the model for age at UKBB entry (age at enrolment) either alongside the binary age of ON diagnosis or instead of it resulted in significant improvement of the model. This resulted in reduction of the *P* value of MS-GRS in the model to less than 0.005, and the log-likelihood ratio test, when comparing the full model to that without MS-GRS was reduced. However, we decided to include in our analysis only those variables that would be available in other cohorts and that would not be specific to UKBB.

3.2. Summary of other subgroup analyses

We performed main analysis, including MS-GRS validation using ROC-AUC on MS cases vs the rest and Cox proportional hazard model of MS-free survival in undifferentiated ON, using two rigid exclusion criteria separately: excluding early diagnoses and excluding self-reported and/or unverified first-occurrence diagnoses.

3.2.1. Excluding cases diagnosed before 20 years of age

26 cases of MS and 19 cases of ON were diagnosed before 20 years of age. Excluding these cases resulted in removal of 22 MS cases, 16 ON cases and 7 MS-ON, bringing the numbers to 2081, 405 and 259 respectively.

3.2.2. Strict definition of diagnoses

We performed main analysis including only cases obtained from either Hospital Episode Statistics (HES) or primary-care data (*See* **Supplementary Fig. 1**). These excluded cases coming only from self-reports or first-occurrence. This resulted in exclusion of 217 MS only, 104 ON only and 36 MS-ON cases, bringing the total number of cases to 1887, 317 and 230 for MS, ON and MS-ON respectively.

3.2.3. Summary and comparison of subgroup analyses

Summary of main analysis in all subgroups is provided in **Supplementary Table 12**. MS-GRS was comparably discriminative of MS cases in all subgroups, both on its own and when combined with covariates, as shown by ROC-AUC after cross-validation. Furthermore, in Cox predictive analysis of future MS in undifferentiated ON, MS-GRS remained significant in all subgroups, and HR per standard deviation of MS-GRS was comparable (**Supplementary Table 12**).

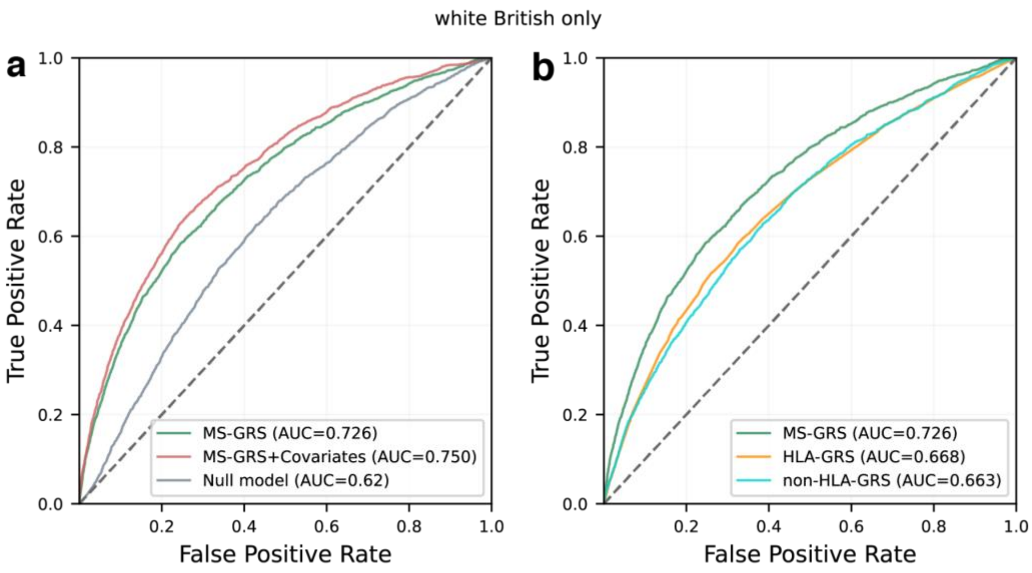

Supplementary Fig. 19 summarises performance of MS-GRS in white British only population **A** ROC-AUC of MS-GRS and covariates. **B** ROC-AUC curves of non-HLA- and HLA-GRS.

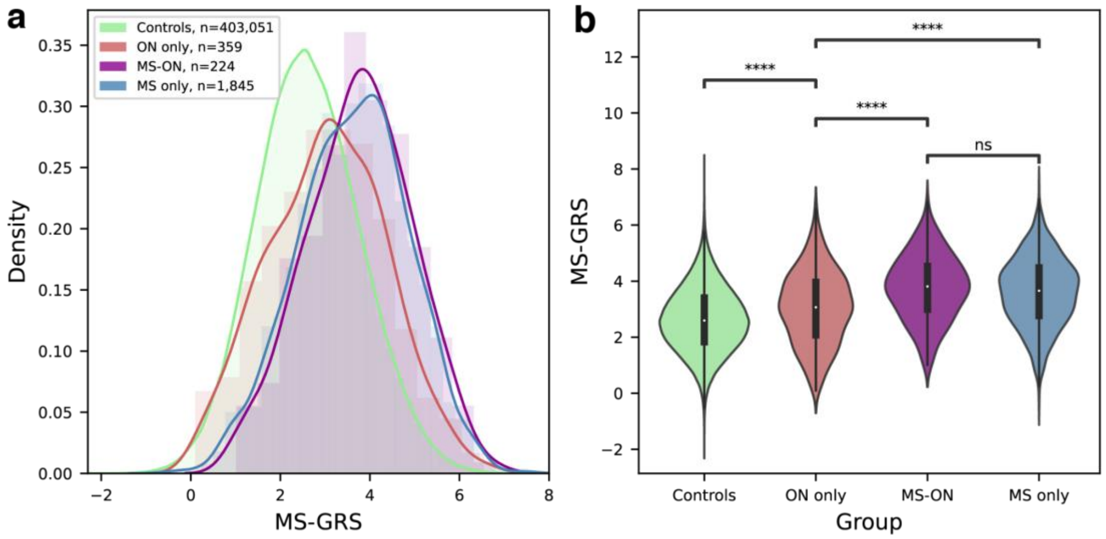

Supplementary Fig. 20 illustrates MS-GRS distribution graphs comparing controls, undifferentiated ON, MS-ON and MS without ON in white British only. **A** shows a histogram plot with kernel density estimate (solid lines). **B** shows a violin plot with Welch's t-test statistical significance: **** – $P < 1.00e\text{-}04$, ns – not statistically significant at $P > 0.05$

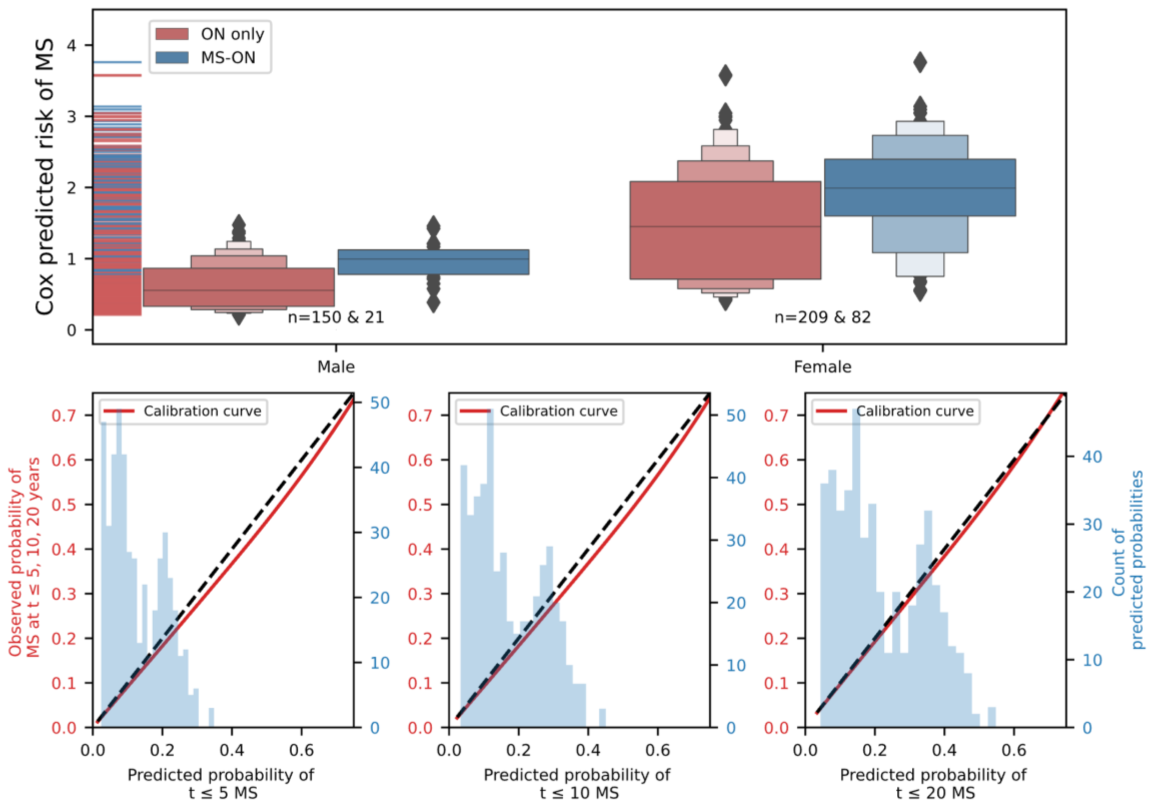

Supplementary Fig. 21 shows a boxen plot illustrating predicted risk of MS in undifferentiated ON stratified by sex, showing that predicted risk is higher in MS-ON cases in both males and females. Risk predicted by Cox proportional hazard model based on sex, age of ON diagnosis [18 to 50] and MS-GRS. Panels **b-d** illustrate calibration plots of the Cox model at three points in time (5,10, and 20 years, respectively). Smoothed calibration curve is shown in red, and ideal calibration in black dotted line.

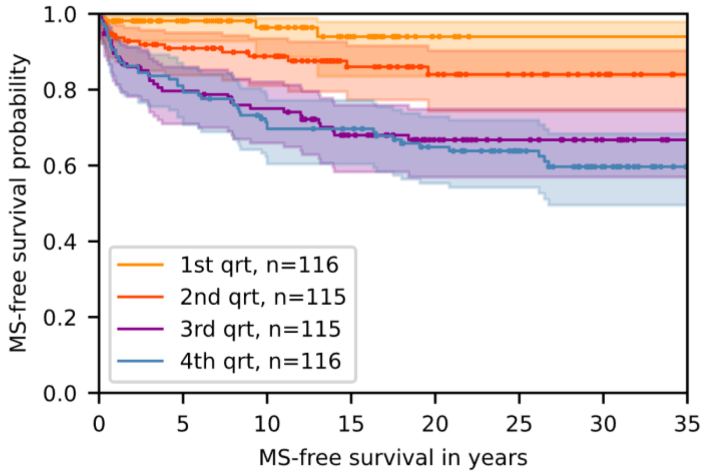

	0	5	10	15	20	25	30	35
**1st qrt, n=116**								
At risk	116	71	49	33	17	13	13	13
Censored	0	43	64	79	95	99	99	99
Events	0	2	3	4	4	4	4	4
**2nd qrt, n=115**								
At risk	115	94	80	53	41	34	26	19
Censored	0	11	23	48	59	66	74	81
Events	0	10	12	14	15	15	15	15
**3rd qrt, n=115**								
At risk	115	88	80	61	46	35	26	17
Censored	0	4	7	19	33	44	53	62
Events	0	23	28	35	36	36	36	36
**4th qrt, n=116**								
At risk	116	92	78	76	63	50	33	19
Censored	0	0	3	5	13	25	39	53
Events	0	24	35	35	40	41	44	44

Supplementary Fig. 22. Kaplan-Meier plot of undifferentiated ON stratified by quartiles of predicted MS risk.

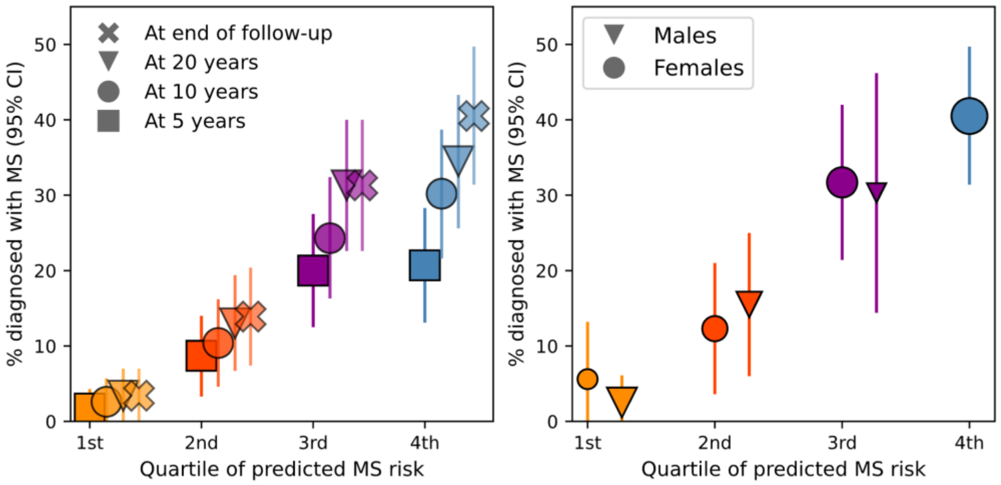

Supplementary Fig. 23 summarises proportion of MS-ON out of all initially undifferentiated ON **A.** Shows at different time points following ON diagnosis in each quartile of predicted MS risk at 5 years, 10 years, 20 years and at the end of follow-up (squares, circles triangles and crosses, respectively)**. B.** Shows proportion of MS-ON at the end of follow-up (median 22.0, IQR 13-34) for males and females (triangles and circles, respectively) in each quartile of predicted MS risk, with the marker size proportional to the proportion of that sex within each quartile.

Supplementary Table 12. Summary of statistical analysis in all subgroups, including all the UKBB, white British only, excluding early cases, and strict definition of diagnoses. All *P*-values not marked with an aster are derived from a from multivariate Cox MS-free survival model with binary age at ON diagnosis, sex, and MS-GRS in that population; *Partial Log-likelihood ratio (LLR) test, comparing models with MS-GRS, sex, and age at ON diagnosis [18-50] to a null model (sex and age binary at ON diagnosis).

**Population**	**Whole of UKBB**	**European ancestry British**	**Non-European ancestry-British**	**Strict diagnoses**
**Controls, n**	480 690	403,051	77,639	480,690
**MS only, n**	2,103	1,845	258	1,887
**ON only, n**	421	359	62	317
**MS-ON, n**	266	224	42	230
**MS-GRS ROC-AUC, 95% CI**	0.721 (0.718-0.723)	0.726 (0.723-0.728)	0.696 (0.688-0.703)	0.721 (0.717-0.725)
**MS-GRS & Covariates ROC-AUC, 95% CI**	0.752 (0.750-0.755)	0.749 (0.747-0.752)	0.753 (0.746 - 0.760)	0.751 (0.748-0.753)
**n undifferentiated ON, no MS (F:M ratio)**	421 (1.49)	359 (1.39)	62 (2.38)	317 (1.52)
**N undifferentiated ON, progressed to MS (F:M ratio)**	124 (4.17)	103 (3.90)	21 (6)	106 (4.58)
**HR of female sex, 95% CI**	2.20 (1.41-3.45), *P*=0.0005	2.18 (1.34-3.52), *P*=0.0016	1.81 (0.53-6.24), *P*=0.3464	2.1 (1.28-3.46), *P*=0.0035
**HR of ON diagnosed between 18 and 50, 95% CI**	2.43 (1.41-4.17), P=0.0014	2.52 (1.52-4.58), *P*=0.0028	2.47 (0.69-8.77), P=0.1627	2.14 (1.2-3.82), P=0.0104
**HR of MS-GRS per SD, 95% CI**	1.29 (1.07-1.55), P=0.0067	1.29 (1.05-1.58), *P*=0.0158	1.4 (0.89-2.22), P=0.1501	1.27 (1.04-1.55), P=0.0188
**Concordance**	0.67	0.65	0.69	0.64
**LLR test *P*-value***	0.0063	0.0151	0.1390	0.0182
**partial AIC**	1405.39	1147.43	147.05	1147.89

4. Full list of Consortia members

4.1. UK Biobank Eye Health & Vision Consortium

Naomi Allen[1], Tariq Aslam[2], Denize Atan[3], Konstantinos Balaskas[4], Sarah Barman[5], Jenny Barrett[6], Paul Bishop[2], Graeme Black[2], Tasanee Braithwaite[7], Roxana Carare[8], Usha Chakravarthy[9], Michelle Chan[4], Sharon Chua[10], Alexander Day[4], Parul Desai[4], Bal Dhillon[11], Andrew Dick[3], Alexander Doney[12], Cathy Egan [4], Sarah Ennis[8], Paul Foster[10], Marcus Fruttiger[10], John Gallacher[1], David (Ted) Garway-Heath[10], Jane Gibson[8], Jeremy Guggenheim[13], Chris Hammond[14], Alison Hardcastle[10], Simon Harding[15], Ruth Hogg[9], Pirro Hysi [14], Pearse Keane[10], Sir Peng Tee Khaw[10], Anthony Khawaja[4], Gerassimos Lascaratos[4], Thomas Littlejohns[1], Andrew Lotery[8], Robert Luben[10], Phil Luthert[10], Tom MacGillivray [11], Sarah Mackie[6], Savita Madhusudhan[16], Bernadette McGuinness[9], Gareth McKay[9], Martin McKibbin [17], Tony Moore[10], James Morgan[13], Eoin O'Sullivan [18], Richard Oram[19], Chris Owen[20], Praveen Patel[4], Euan Paterson[9], Tunde Peto[9], Axel Petzold[21], Nikolas Pontikos[10], Jugnoo Rahi [22], Alicja Rudnicka [20], Naveed Sattar[23], Jay Self[8], Panagiotis Sergouniotis[2], Sobha Sivaprasad[4], David Steel[24], Irene Stratton [25], Nicholas Strouthidis[4], Cathie Sudlow[11], Zihan Sun[10], Robyn Tapp[20], Dhanes Thomas [4], Emanuele Trucco[12], Adnan Tufail[4], Ananth Viswanathan[4], Veronique Vitart[11], Mike Weedon[19], Katie Williams[14], Cathy Williams[3], Jayne Woodside[9], Max Yates[26], Yalin Zheng[15]

1. University of Oxford, Oxford, England, United Kingdom
2. The University of Manchester, Manchester, England, United Kingdom
3. University of Bristol, Bristol, England, United Kingdom
4. Moorfields Eye Hospital, London, England, United Kingdom
5. Kingston University, London, England, United Kingdom
6. University of Leeds, Leeds, West Yorkshire, United Kingdom
7. St Thomas' Hospital, London, England, United Kingdom
8. University of Southampton, Southampton, England, United Kingdom
9. Queen's University Belfast, Belfast, Northern Ireland, United Kingdom
10. UCL Institute of Ophthalmology, London, England, United Kingdom
11. University of Edinburgh, Edinburgh, Scotland, United Kingdom
12. University of Dundee, Dundee, Scotland, United Kingdom
13. Cardiff University, Cardiff, Wales, United Kingdom
14. King's College London, London, England, United Kingdom
15. University of Liverpool, Liverpool, England, United Kingdom
16. Royal Liverpool University Hospital, Liverpool, England, United Kingdom
17. Leeds Teaching Hospitals NHS Trust, Leeds, West Yorkshire, United Kingdom
18. King's College Hospital, London, England, United Kingdom
19. University of Exeter, Exeter, England, United Kingdom
20. St George's, University of London, London, England, United Kingdom
21. UCL Institute of Neurology, London, England, United Kingdom
22. UCL Institute of Child Health, London, England, United Kingdom
23. University of Glasgow, Glasgow, Scotland, United Kingdom
24. Newcastle University, Newcastle, England, United Kingdom
25. Gloucestershire Hospitals NHS Foundation Trust, Gloucester, England, United Kingdom
26. University of East Anglia, Norwich, England, United Kingdom

4.2. Geisinger-Regeneron DiscovEHR

Adam Buchanan[27], David J. Carey[27], Christa L. Martin[27], Michelle Meyer[27], Kyle Retterer[27], David Rolston[27], Goncalo Abecasis[28], Aris Baras[28], Michael Cantor[28], Giovanni Coppola[28], Aris Economides[28], Luca A. Lotta[28], John D. Overton[28], Jeffrey G. Reid[28], Alan Shuldiner[28], Katia Karalis[28], Katherine Siminovitch[28], Christina Beechert[28], Caitlin Forsythe[28], Erin D. Fuller[28], Zhenhua Gu[28], Michael Lattari[28], Alexander Lopez[28], John D. Overton[28], Thomas D. Schleicher[28], Maria Sotiropoulos Padilla[28], Louis Widom[28], Sarah E. Wolf[28], Manasi Pradhan[28], Kia Manoochehri[28], Ricardo H. Ulloa.[28], Xiaodong Bai[28], Suganthi Balasubramanian[28], Andrew Blumenfeld[28], Boris Boutkov[28], Gisu Eom[28], Lukas Habegger[28], Alicia Hawes[28], Shareef Khalid[28], Olga Krasheninina[28], Rouel Lanche[28], Adam J. Mansfield[28], Evan K. Maxwell[28], Mrunali Nafde[28], Sean O'Keeffe[28], Max Orelus[28], Razvan Panea[28], Tommy Polanco[28], Ayesha Rasool[28], Jeffrey G. Reid[28], William Salerno[28], Jeffrey C. Staples[28], Michael Cantor[28], Dadong Li[28], Deepika Sharma[28], Marcus B. Jones[28], Jason Mighty[28], Lyndon J. Mitnaul[28]

27. Geisinger, Danville, PA, USA
28. Regeneron Genetics Center, Tarrytown, NY, USA

4.3. FinnGen

Aarno Palotie[29], Mark Daly[29], Bridget Riley-Gills[30], Howard Jacob[30], Dirk Paul[31], Slavé Petrovski[31], Heiko Runz[32], Sally John[32], George Okafo[33], Robert Plenge[34], Joseph Maranville[34], Mark McCarthy[35], Margaret G. Ehm[36], Kirsi Auro[37], Simonne Longerich[38], Anders Mälarstig[39], Katherine Klinger[40], Clement Chatelain[40], Matthias Gossel[40], Karol Estrada[41], Robert Graham[41], Dawn Waterworth[42], Chris O´Donnell[43], Nicole Renaud[43], Tomi P. Mäkelä[44], Jaakko Kaprio[29], Petri Virolainen[45], Antti Hakanen[45], Terhi Kilpi[46], Markus Perola[46], Jukka Partanen[47], Anne Pitkäranta[48], Taneli Raivio[48], Jani Tikkanen[49], Raisa Serpi[49], Tarja Laitinen[50], Veli-Matti Kosma[51], Jari Laukkanen[52], Marco Hautalahti[53], Outi Tuovila[54], Raimo Pakkanen[54], Jeffrey Waring[30], Bridget Riley-Gillis[30], Fedik Rahimov[30], Ioanna Tachmazidou[31], Chia-Yen Chen[32], Zhihao Ding[33], Marc Jung[33], Hanati Tuoken[33], Shameek Biswas[34], Rion Pendergrass[35], David Pulford[55], Neha Raghavan[38], Adriana Huertas-Vazquez[38], Jae-Hoon Sul[38], Xinli Hu[39], Åsa Hedman[39], Manuel Rivas[41], Ma´en Obeidat[43], Jonathan Chung[43], Jonas Zierer[43], Mari Niemi[43], Samuli Ripatti[29], Johanna Schleutker[56], Mikko Arvas[47], Olli Carpén[48], Reetta Hinttala[49], Johannes Kettunen[49], Arto Mannermaa[51], Katriina Aalto-Setälä[57], Mika Kähönen[50], Johanna Mäkelä[53], Reetta Kälviäinen[58], Valtteri Julkunen[58], Hilkka Soininen[58], Anne Remes[59], Mikko Hiltunen[60], Jukka Peltola[61], Minna Raivio[62], Pentti Tienari[62], Juha Rinne[63], Roosa Kallionpää[63], Juulia Partanen[64], Adam Ziemann[30], Nizar Smaoui[30], Anne Lehtonen[30], Susan Eaton[32], Sanni Lahdenperä[32], Natalie Bowers[35], Edmond Teng[35], Fanli Xu[65], Laura Addis[65], John Eicher[65], Qingqin S Li[66], Karen He[42], Ekaterina Khramtsova[42], Martti Färkkilä[62], Jukka Koskela[62], Sampsa Pikkarainen[62], Airi Jussila[61], Katri Kaukinen[61], Timo Blomster[59], Mikko Kiviniemi[58], Markku Voutilainen[63], Tim Lu[35], Linda McCarthy[65], Amy Hart[42], Meijian Guan[42], Jason Miller[38], Kirsi Kalpala[39], Melissa Miller[39], Kari Eklund[62], Antti Palomäki[63], Pia Isomäki[61], Laura Pirilä[63], Oili Kaipiainen-Seppänen[58], Johanna Huhtakangas[59], Nina Mars[29], Apinya Lertratanakul[30], Coralie Viollet[67], Marla Hochfeld[34], Jorge Esparza Gordillo[65], Fabiana Farias[38], Nan Bing[39], Margit Pelkonen[58], Paula Kauppi[62],

Hannu Kankaanranta[68], Terttu Harju[59], Riitta Lahesmaa[63], Hubert Chen[35], Joanna Betts[65], Rajashree Mishra[65], Majd Mouded[69], Debby Ngo[69], Teemu Niiranen[70], Felix Vaura[70], Veikko Salomaa[70], Kaj Metsärinne[63], Jenni Aittokallio[63], Jussi Hernesniemi[61], Daniel Gordin[62], Juha Sinisalo[62], Marja-Riitta Taskinen[62], Tiinamaija Tuomi[62], Timo Hiltunen[62], Amanda Elliott[29], Mary Pat Reeve[29], Sanni Ruotsalainen[29], Audrey Chu[65], Dermot Reilly[71], Mike Mendelson[72], Jaakko Parkkinen[39], Tuomo Meretoja[62], Heikki Joensuu[62], Johanna Mattson[62], Eveliina Salminen[62], Annika Auranen[73], Peeter Karihtala[59], Päivi Auvinen[58], Klaus Elenius[63], Esa Pitkänen[29], Relja Popovic[30], Margarete Fabre[67], Jennifer Schutzman[35], Diptee Kulkarni[65], Alessandro Porello[42], Andrey Loboda[38], Heli Lehtonen[39], Stefan McDonough[39], Sauli Vuoti[74], Kai Kaarniranta[58], Joni A Turunen[75], Terhi Ollila[62], Hannu Uusitalo[61], Juha Karjalainen[29], Mengzhen Liu[30], Stephanie Loomis[32], Erich Strauss[35], Hao Chen[35], Kaisa Tasanen[59], Laura Huilaja[59], Katariina Hannula-Jouppi[62], Teea Salmi[61], Sirkku Peltonen[63], Leena Koulu[63], David Choy[35], Ying Wu[39], Pirkko Pussinen[62], Aino Salminen[62], Tuula Salo[62], David Rice[62], Pekka Nieminen[62], Ulla Palotie[62], Maria Siponen[58], Liisa Suominen[58], Päivi Mäntylä[58], Ulvi Gursoy[63], Vuokko Anttonen[59], Kirsi Sipilä[76], Rion Pendergrass [35], Hannele Laivuori[29], Venla Kurra[61], Laura Kotaniemi-Talonen[61], Oskari Heikinheimo[62], Ilkka Kalliala[62], Lauri Aaltonen[62], Varpu Jokimaa[63], Marja Vääräsmäki[59], Outi Uimari[59], Laure Morin-Papunen[59], Maarit Niinimäki[59], Terhi Piltonen[59], Katja Kivinen[29], Elisabeth Widen[29], Taru Tukiainen[29], Niko Välimäki[77], Eija Laakkonen[78], Jaakko Tyrmi[79], Heidi Silven[79], Eeva Sliz[79], Riikka Arffman[79], Susanna Savukoski[79], Triin Laisk[80], Natalia Pujol[80], Janet Kumar[36], Iiris Hovatta[81], Erkki Isometsä[62], Hanna Ollila[29], Jaana Suvisaari[70], Thomas Damm Als[82], Antti Mäkitie[83], Argyro Bizaki-Vallaskangas[61], Sanna Toppila-Salmi[84], Tytti Willberg[63], Elmo Saarentaus[29], Antti Aarnisalo[62], Elisa Rahikkala[59], Kristiina Aittomäki[85], Fredrik Åberg[86], Mitja Kurki[29], Aki Havulinna[29], Juha Mehtonen[29], Priit Palta[29], Shabbeer Hassan[29], Pietro Della Briotta Parolo[29], Wei Zhou[87], Mutaamba Maasha[87], Susanna Lemmelä[29], Aoxing Liu[29], Arto Lehisto[29], Andrea Ganna[29], Vincent Llorens[29], Henrike Heyne[29], Joel Rämö[29], Rodos Rodosthenous[29], Satu Strausz[29], Tuula Palotie[88], Kimmo Palin[77], Javier Garcia-Tabuenca[89], Harri Siirtola[89], Tuomo Kiiskinen[29], Jiwoo Lee[29], Kristin Tsuo[29], Kati Kristiansson[46], Kati Hyvärinen[90], Jarmo Ritari[90], Katri Pylkäs[79], Minna Karjalainen[79], Tuomo Mantere[49], Eeva Kangasniemi[50], Sami Heikkinen[60], Nina Pitkänen[45], Samuel Lessard[40], Clément Chatelain[40], Lila Kallio[45], Tiina Wahlfors[46], Eero Punkka[48], Sanna Siltanen[50], Teijo Kuopio[52], Anu Jalanko[29], Huei-Yi Shen[29], Risto Kajanne[29], Mervi Aavikko[29], Helen Cooper[29], Denise Öller[29], Rasko Leinonen[29], Henna Palin[50], Malla-Maria Linna[48], Masahiro Kanai[87], Zhili Zheng[87], L. Elisa Lahtela[29], Mari Kaunisto[29], Elina Kilpeläinen[29], Timo P. Sipilä[29], Oluwaseun Alexander Dada[29], Awaisa Ghazal[29], Anastasia Kytölä[29], Rigbe Weldatsadik[29], Kati Donner[29], Anu Loukola[48], Päivi Laiho[46], Tuuli Sistonen[46], Essi Kaiharju[46], Markku Laukkanen[46], Elina Järvensivu[46], Sini Lähteenmäki[46], Lotta Männikkö[46], Regis Wong[46], Auli Toivola[46], Minna Brunfeldt[46], Hannele Mattsson[46], Sami Koskelainen[46], Tero Hiekkalinna[46], Teemu Paajanen[46], Shuang Luo[29], Shanmukha Sampath Padmanabhuni[29], Marianna Niemi[89], Javier Gracia-Tabuenca[89], Mika Helminen[89], Tiina Luukkaala[89], Iida Vähätalo[89], Jyrki Tammerluoto[29], Sarah Smith[91], Tom Southerington[91], Petri Lehto[91]

29. Institute for Molecular Medicine Finland (FIMM), HiLIFE, University of Helsinki, Helsinki, Finland
30. Abbvie, Chicago, IL, United States

31. Astra Zeneca, Cambridge, United Kingdom
32. Biogen, Cambridge, MA, United States
33. Boehringer Ingelheim, Ingelheim am Rhein, Germany
34. Bristol Myers Squibb, New York, NY, United States
35. Genentech, San Francisco, CA, United States
36. GlaxoSmithKline, Collegeville, PA, United States
37. GlaxoSmithKline, Espoo, Finland
38. Merck, Kenilworth, NJ, United States
39. Pfizer, New York, NY, United States
40. Translational Sciences, Sanofi R&D, Framingham, MA, USA
41. Maze Therapeutics, San Francisco, CA, United States
42. Janssen Research & Development, LLC, Spring House, PA, United States
43. Novartis Institutes for BioMedical Research, Cambridge, MA, United States
44. HiLIFE, University of Helsinki, Finland, Finland
45. Auria Biobank, University of Turku, Hospital District of Southwest Finland, Turku, Finland
46. THL Biobank, Finnish Institute for Health and Welfare (THL), Helsinki, Finland
47. Finnish Red Cross Blood Service, Finnish Hematology Registry and Clinical Biobank, Helsinki, Finland
48. Helsinki Biobank, Helsinki University and Hospital District of Helsinki and Uusimaa, Helsinki
49. Northern Finland Biobank Borealis, University of Oulu, Northern Ostrobothnia Hospital District, Oulu, Finland
50. Finnish Clinical Biobank Tampere, University of Tampere, Pirkanmaa Hospital District, Tampere, Finland
51. Biobank of Eastern Finland, University of Eastern Finland, Northern Savo Hospital District, Kuopio, Finland
52. Central Finland Biobank, University of Jyväskylä, Central Finland Health Care District, Jyväskylä, Finland
53. FINBB - Finnish biobank cooperative
54. Business Finland, Helsinki, Finland
55. GlaxoSmithKline, Stevenage, United Kingdom
56. Auria Biobank, Hospital District of Southwest Finland, Turku, Finland
57. Faculty of Medicine and Health Technology, Tampere University, Tampere, Finland
58. Northern Savo Hospital District, Kuopio, Finland
59. Northern Ostrobothnia Hospital District, Oulu, Finland
60. University of Eastern Finland, Kuopio, Finland
61. Pirkanmaa Hospital District, Tampere, Finland
62. Hospital District of Helsinki and Uusimaa, Helsinki, Finland
63. Hospital District of Southwest Finland, Turku, Finland
64. Institute for Molecular Medicine Finland, HiLIFE, University of Helsinki, Finland
65. GlaxoSmithKline, Brentford, United Kingdom
66. Janssen Research & Development, LLC, Titusville, NJ 08560, United States
67. AstraZeneca, Cambridge, United Kingdom
68. University of Gothenburg, Gothenburg, Sweden
69. Novartis, Basel, Switzerland
70. Finnish Institute for Health and Welfare (THL), Helsinki, Finland

71. Janssen Research & Development, LLC, Boston, MA, United States
72. Novartis, Boston, MA, United States
73. Pirkanmaa Hospital District , Tampere, Finland
74. Janssen-Cilag Oy, Espoo, Finland
75. Helsinki University Hospital and University of Helsinki, Helsinki, Finland
76. Research Unit of Oral Health Sciences Faculty of Medicine, University of Oulu, Oulu, Finland
77. University of Helsinki, Helsinki, Finland
78. University of Jyväskylä, Jyväskylä, Finland
79. University of Oulu, Oulu, Finland
80. Estonian biobank, Tartu, Estonia
81. University of Helsinki, Finland
82. Aarhus University, Denmark
83. Department of Otorhinolaryngology - Head and Neck Surgery, University of Helsinki and Helsinki University Hospital, Helsinki, Finland
84. University of Eastern Finland and Kuopio University Hospital, Department of Otorhinolaryngology, Kuopio, Finland
85. Department of Medical Genetics, Helsinki University Central Hospital, Helsinki, Finland
86. Transplantation and Liver Surgery Clinic, Helsinki University Hospital, Helsinki University, Helsinki, Finland
87. Broad Institute, Cambridge, MA, United States
88. University of Helsinki and Hospital District of Helsinki and Uusimaa, Helsinki, Finland
89. University of Tampere, Tampere, Finland
90. Finnish Red Cross Blood Service, Helsinki, Finland
91. Finnish Biobank Cooperative (FINBB), Turku, Finland

References

1. Biobabank, U. Health Outcomes Overview.
2. Sudlow, C., *et al.* UK biobank: an open access resource for identifying the causes of a wide range of complex diseases of middle and old age. *PLoS Med* **12**, e1001779 (2015).
3. International Multiple Sclerosis Genetics, C. Multiple sclerosis genomic map implicates peripheral immune cells and microglia in susceptibility. *Science* **365**(2019).
4. Bycroft, C., *et al.* The UK Biobank resource with deep phenotyping and genomic data. *Nature* **562**, 203-209 (2018).
5. Moutsianas, L., *et al.* Class II HLA interactions modulate genetic risk for multiple sclerosis. *Nat Genet* **47**, 1107-1113 (2015).
6. Carey, D.J., *et al.* The Geisinger MyCode community health initiative: an electronic health record-linked biobank for precision medicine research. *Genet Med* **18**, 906-913 (2016).
7. Dewey, F.E., *et al.* Distribution and clinical impact of functional variants in 50,726 whole-exome sequences from the DiscovEHR study. *Science* **354**(2016).
8. Collins, G.S., Reitsma, J.B., Altman, D.G. & Moons, K.G. Transparent reporting of a multivariable prediction model for individual prognosis or diagnosis (TRIPOD): the TRIPOD Statement. *BMC Med* **13**, 1 (2015).
9. Luo, Y., *et al.* A high-resolution HLA reference panel capturing global population diversity enables multi-ancestry fine-mapping in HIV host response. *Nat Genet* **53**, 1504-1516 (2021).
10. Jia, X., *et al.* Imputing amino acid polymorphisms in human leukocyte antigens. *PLoS One* **8**, e64683 (2013).
11. Staples, J., *et al.* Profiling and Leveraging Relatedness in a Precision Medicine Cohort of 92,455 Exomes. *Am J Hum Genet* **102**, 874-889 (2018).
12. FinnGen, I.f.M.M.F.F., University of Helsinki. FINNGEN RESEARCH PROJECT. Vol. 2023 (2023).
13. Kurki, M.I., *et al.* FinnGen provides genetic insights from a well-phenotyped isolated population. *Nature* **613**, 508-518 (2023).
14. Ritari, J., *et al.* Increasing accuracy of HLA imputation by a population-specific reference panel in a FinnGen biobank cohort. *NAR Genom Bioinform* **2**, lqaa030 (2020).
15. McKinney, W.a.o. Data structures for statistical computing in python. *Proceedings of the 9th Python in Science Conference* **445**, 51-56 (2010).
16. Harris, C.R., *et al.* Array programming with NumPy. *Nature* **585**, 357-362 (2020).
