# Supplementary material for: Applying a genetic risk score model to enhance prediction of future multiple sclerosis diagnosis at first presentation with optic neuritis
Source: Nat Commun. 2024 Feb 28;15:1415. doi: 10.1038/s41467-024-44917-9 (PMC10902342; doi:10.1038/s41467-024-44917-9)
Supplement: Supplementary file 3 — Description of Additional Supplementary Files [file 41467_2024_44917_MOESM3_ESM.pdf]

Description of Additional Supplementary Files

File Name: Supplementary Data 1
Description: Table of single nucleotide polymorphisms used in the construction of the genetic risk score with allele frequencies and imputation scores. This file is referred to in the methods and supplementary methods sections.
