# Supplementary material for: Applying a genetic risk score model to enhance prediction of future multiple sclerosis diagnosis at first presentation with optic neuritis
Source: Nat Commun. 2024 Feb 28;15:1415. doi: 10.1038/s41467-024-44917-9 (PMC10902342; doi:10.1038/s41467-024-44917-9)
Supplement: Supplementary file 5 — Reporting Summary [file 41467_2024_44917_MOESM5_ESM.pdf]

nature portfolio

Corresponding author(s): Richard Oram

Last updated by author(s): Dec 20, 2023

Reporting Summary

Nature Portfolio wishes to improve the reproducibility of the work that we publish. This form provides structure for consistency and transparency in reporting. For further information on Nature Portfolio policies, see our Editorial Policies and the Editorial Policy Checklist.

Statistics

For all statistical analyses, confirm that the following items are present in the figure legend, table legend, main text, or Methods section.

n/a	Confirmed	
☐	☒	The exact sample size (*n*) for each experimental group/condition, given as a discrete number and unit of measurement
☐	☒	A statement on whether measurements were taken from distinct samples or whether the same sample was measured repeatedly
☐	☒	The statistical test(s) used AND whether they are one- or two-sided<br>*Only common tests should be described solely by name; describe more complex techniques in the Methods section.*
☐	☒	A description of all covariates tested
☒	☐	A description of any assumptions or corrections, such as tests of normality and adjustment for multiple comparisons
☐	☒	A full description of the statistical parameters including central tendency (e.g. means) or other basic estimates (e.g. regression coefficient) AND variation (e.g. standard deviation) or associated estimates of uncertainty (e.g. confidence intervals)
☐	☒	For null hypothesis testing, the test statistic (e.g. *F*, *t*, *r*) with confidence intervals, effect sizes, degrees of freedom and *P* value noted<br>*Give P values as exact values whenever suitable.*
☒	☐	For Bayesian analysis, information on the choice of priors and Markov chain Monte Carlo settings
☒	☐	For hierarchical and complex designs, identification of the appropriate level for tests and full reporting of outcomes
☒	☐	Estimates of effect sizes (e.g. Cohen's *d*, Pearson's *r*), indicating how they were calculated

Our web collection on statistics for biologists contains articles on many of the points above.

Software and code

Policy information about availability of computer code

Data collection	No primary data was collected in this study.
Data analysis	The code used for phenotype, genotype, and statistical analysis is available through the following GitHub repository: https://github.com/ploginovic/MS-ON-ukb-code. Statistical analyses were performed in Python v3.10 (https://docs.python.org/release/3.10.11/), with adaptations the LifeLines Python package, covered by the MIT license (https://github.com/CamDavidsonPilon/lifelines). Genetic analyses were performed in PLINK v1.9 (https://www.cog-genomics.org/plink/) and PLINK v2.0.a (https://www.cog-genomics.org/plink/2.0/). Phenotype analyses were performed in STATA v17 (https://www.stata.com) and R v3.6 (https://www.r-project.org).

For manuscripts utilizing custom algorithms or software that are central to the research but not yet described in published literature, software must be made available to editors and reviewers. We strongly encourage code deposition in a community repository (e.g. GitHub). See the Nature Portfolio guidelines for submitting code & software for further information.

Data

Policy information about availability of data

All manuscripts must include a data availability statement. This statement should provide the following information, where applicable:

- Accession codes, unique identifiers, or web links for publicly available datasets
- A description of any restrictions on data availability
- For clinical datasets or third party data, please ensure that the statement adheres to our policy

DATA AVAILABILITY STATEMENT
The Genetic risk score will be deposited in the Polygenic Score Catalog (PGS Catalog: https://www.pgscatalog.org/) upon receiving a DOI of this study. Individual-level genotype data described in this study are available to bona fide researchers as per the UK Biobank data-access protocol (https://www.ukbiobank.ac.uk/enable-your-research/apply-for-access). Further details and instructions about registration for access to the data are available at http://www.ukbiobank.ac.uk/register-apply/. UK Biobank accession codes of this study were 9055 and 9072. For FinnGen data, access to individual-level sensitive health data must be approved by national authorities for specific research projects and for specifically listed and approved researchers in accordance with the National and European regulations (GDPR). Researchers can apply for the health register data from the Finnish Data Authority Findata (https://findata.fi/en/permits/) and for individual-level genotype data from Finnish biobanks via the Fingenious portal (https://site.fingenious.fi/en/) hosted by the Finnish Biobank Cooperative FINBB (https://finbb.fi/en/). For Geisinger, the data was generated as described in Carey et al. (Reference 58). Further details regarding phenotype and genotyping data for Geisinger can be found here: https://www.geisinger.org/precision-health/mycode/discovehr-project. Institutional Review Board determined this study to be “Non-human subject research” using de-identified information (IRB #: 2023-1075). The HLA genotyping data and MS-GRS from the MyCode participants in this study may be shared with third party bona fide researchers upon execution of the data-sharing agreement.

Human research participants

Policy information about studies involving human research participants and Sex and Gender in Research.

Reporting on sex and gender	We used self-reported sex as a covariate in calculating the MS-GRS in UK Bioabank, FinnGen and Geisigner, and reported across three cohorts. In the longitudinal analysis, we used genetic sex, and where it was missing, we used reported sex, in developing (UKBB) and validating the models (Geisinger, FinnGen).
Population characteristics	Detailed population characteristics are provided in Table 1 in the manuscript and Supplementary Table 5 in the Supplementary Information.
Recruitment	N/A
Ethics oversight	UKBB participants gave informed consent to participate, and ethics committee approval was granted by the Northwest Multi-Centre Research Ethics Committee (ref 06/MRE08/65).The Geisinger Institutional Review Board determined this study to be “Non-human subject research” using de-identified information (IRB #: 2023-1075). The FinnGen study protocol (Nr HUS/990/2017) is approved by the Coordinating Ethics Committee of the Hospital District of Helsinki and Uusimaa (HUS). or regarding earlier cohorts, with approval from Fimea, the National Supervisory Authority for Welfare and Health. The FinnGen study protocol (number HUS/990/2017) is approved by the Coordinating Ethics Committee of the Hospital District of Helsinki and Uusimaa (HUS).

Note that full information on the approval of the study protocol must also be provided in the manuscript.

Field-specific reporting

Please select the one below that is the best fit for your research. If you are not sure, read the appropriate sections before making your selection.

☒ Life sciences ☐ Behavioural & social sciences ☐ Ecological, evolutionary & environmental sciences

For a reference copy of the document with all sections, see nature.com/documents/nr-reporting-summary-flat.pdf

Life sciences study design

All studies must disclose on these points even when the disclosure is negative.

Sample size	Sample size was determined by the data available in UK Biobank (UKBB), as well as data from FinnGen and Geisinger cohorts.<br><br>In UKBB, from 483,506 unrelated individuals with available genetic and phenotype data, of whom 83.9% were of European ancestry, we identified 2,369 multiple sclerosis (MS) cases and 687 optic neuritis (ON) cases. ON cases included 545 (545 out of 687, 79.3%) who were not known to have MS at first ON presentation and 142 (20.7%) with prior or simultaneous diagnosis of MS (MS-ON). During cumulative follow-up from first ON presentation to latest data extraction in 2019 or death (median 18.4 years, IQR 9.9-30.2) a further 124 out of 545 (22.8%) were diagnosed with MS.<br><br>We analysed data from 169,762 individuals in the Geisinger DiscovEHR. Only unrelated individuals up to third-degree relationship (n = 116,767) from the MyCode cohort were included in study. We identified people with multiple sclerosis without opitc neuritis (n = 1901),

people with MS-ON (n=420), people with ON without MS (n = 695), and healthy controls (n = 113,751). For UKBB model validation, we used 835 individuals with undifferentiated optic neuritis, from which 140 developed multiple sclerosis subsequently.

In FinnGen, the data was pre-processed by FinnGen analytical team considering HWE and data quality. After removing individuals with cryptic relatedness and individuals without clear demographic information (e.g. sex, age at DNA sample collection), our final study population consisted of 372,416 individuals. Based on diagnostic codes available, we identified 369,633 as healthy controls, 608 individuals with ON without MS, 631 MS-ON individuals, and 1,544 individuals with MS without ON.

Data exclusions	Data exclusion involved genotype and phenotype quality control for missing data and genetic relatedness. Furthermore, we excluded participants based on ambiguous diagnostic codes, described in detail in the manuscript and Supplemental Information. No exclusions were made based on genetic ancestry, and a sensitivity analysis in UKBB is provided in Supplement Information.
Replication	We replicated the UKBB findings in two external cohorts, namely Geisinger (Pennsylvania, USA) and FinnGen (Finland). Replication consisted of applying a model developed and trained on UK Biobank data to predict multiple sclerosis. Data from all replication attempts are reported.
Randomization	No group allocations were made in this study. Individuals were grouped based on diagnostic codes for multiple sclerosis and optic neuritis.
Blinding	No group allocations were made in this study. In the analysis, individuals were grouped based on diagnostic codes from observational cohorts.

Reporting for specific materials, systems and methods

We require information from authors about some types of materials, experimental systems and methods used in many studies. Here, indicate whether each material, system or method listed is relevant to your study. If you are not sure if a list item applies to your research, read the appropriate section before selecting a response.

Materials & experimental systems

n/a	Involved in the study
☒	☐ Antibodies
☒	☐ Eukaryotic cell lines
☒	☐ Palaeontology and archaeology
☒	☐ Animals and other organisms
☒	☐ Clinical data
☒	☐ Dual use research of concern

Methods

n/a	Involved in the study
☒	☐ ChIP-seq
☒	☐ Flow cytometry
☒	☐ MRI-based neuroimaging
